# Endothelial-specific inhibition of NF-κB enhances functional haematopoiesis

**DOI:** 10.1038/ncomms13829

**Published:** 2016-12-21

**Authors:** Michael G. Poulos, Pradeep Ramalingam, Michael C. Gutkin, Maria Kleppe, Michael Ginsberg, Michael J. P. Crowley, Olivier Elemento, Ross L. Levine, Shahin Rafii, Jan Kitajewski, Matthew B. Greenblatt, Jae-Hyuck Shim, Jason M. Butler

**Affiliations:** 1 Department of Genetic Medicine, Ansary Stem Cell Institute, Weill Cornell Medical College, New York, New York 10021, USA; 2 Department of Surgery, Weill Cornell Medical College, New York, New York 10021, USA; 3 Human Oncology and Pathogenesis Program, Memorial Sloan Kettering Cancer Center, New York, New York 10065, USA; 4 Angiocrine Bioscience, New York, New York 10065, USA; 5 Department of Cardiothoracic Surgery, Weill Cornell Medical College, New York, New York 10065, USA; 6 Department of Physiology, Biophysics and Systems Biology, Weill Cornell Medical College, New York, New York 10065, USA; 7 Neuberger Berman Lung Cancer Center, Weill Cornell Medical Center, New York, New York 10065, USA; 8 HRH Prince Alwaleed Bin Talal Bin Abdulaziz Al-Saud Institute for Computational Biomedicine, Weill Cornell Medical College, New York, New York 10065, USA; 9 Leukemia Service, Department of Medicine, Memorial Sloan Kettering Cancer Center, New York, New York 10065, USA; 10 Center for Epigenetics Research, Memorial Sloan Kettering Cancer Center, New York, New York 10065, USA; 11 Department of Medicine, Division of Regenerative Medicine, Ansary Stem Cell Institute, Weill Cornell Medical College, New York, New York 10065, USA; 12 Department of OB/GYN, Columbia University Medical Center, New York, New York 10032, USA; 13 Department of Pathology, Columbia University Medical Center, New York, New York 10032, USA; 14 Pathology and Laboratory Medicine, Weill Cornell Medical College, New York, New York 10021, USA; 15 Department of Medicine, Division of Rheumatology, University of Massachusetts Medical School, Worcester, Massachusetts 01605, USA

## Abstract

Haematopoietic stem cells (HSCs) reside in distinct niches within the bone marrow (BM) microenvironment, comprised of endothelial cells (ECs) and tightly associated perivascular constituents that regulate haematopoiesis through the expression of paracrine factors. Here we report that the canonical NF-κB pathway in the BM vascular niche is a critical signalling axis that regulates HSC function at steady state and following myelosuppressive insult, in which inhibition of EC NF-κB promotes improved HSC function and pan-haematopoietic recovery. Mice expressing an endothelial-specific dominant negative IκBα cassette under the *Tie2* promoter display a marked increase in HSC activity and self-renewal, while promoting the accelerated recovery of haematopoiesis following myelosuppression, in part through protection of the BM microenvironment following radiation and chemotherapeutic-induced insult. Moreover, transplantation of NF-κB-inhibited BM ECs enhanced haematopoietic recovery and protected mice from pancytopenia-induced death. These findings pave the way for development of niche-specific cellular approaches for the treatment of haematological disorders requiring myelosuppressive regimens.

## Abstract (web summary)

The complex microenvironmental signalling pathways that govern haematopoietic stem cell (HSC) activity remain poorly defined. Here, the authors identify endothelial NF-κB signalling as regulating regenerative HSC function, accelerating haematopoietic recovery following myelosuppressive injury in mice.

Adult haematopoietic stem cells (HSCs) are defined by their ability to undergo self-renewal and maintain the capacity to generate all mature haematopoietic cell types within the blood and immune system^1^,^2^. These unique qualities make the HSC clinically useful in bone marrow (BM) transplantation settings for a wide variety of haematological diseases^3^,^4^. There is a large body of evidence demonstrating a functional interaction between the tissue-specific microenvironment and its resident HSC, which modulates stem cell quiescence, self-renewal and differentiation^5^,^6^. Despite advances in the understanding of HSC biology, the exact intrinsic and extrinsic mechanisms that regulate the balance between self-renewal and lineage-specific differentiation are still unknown^2^. Elucidating the mechanisms utilized by the BM microenvironment to regulate HSC fate aim to improve upon current strategies for the *ex vivo* expansion of transplantable, repopulating HSCs for the treatment of life-threatening pancytopenia associated with chemo-irradiation and to facilitate the development of therapeutic approaches to accelerate the regeneration of the BM niche and the HSC pool *in vivo* following myeloablation.

Endothelial cells have a critical role in regulating haematopoiesis throughout life, from the embryonic emergence of definitive HSCs to supporting haematopoietic homeostasis and regeneration following myeloablative injury^7^,^8^,^9^. However, the comprehensive signalling framework within the endothelial niche that supports HSC maintenance and function are not fully understood^2^,^5^,^10^. Within the adult BM microenvironment, endothelial cells are a critical component of niche-mediated HSC maintenance through expression of pro-haematopoietic paracrine factors, including KITL, CXCL12, and JAGGED1 (refs 9, 11, 12). Additionally, signalling through AKT and MAPK pathways within endothelial cells have been shown to modulate HSC maintenance. AKT-activation endows endothelial cells with the capacity to instructively support HSC self-renewal through the expression of pro-haematopoietic paracrine factors during both homeostatic haematopoiesis and regeneration of the haematopoietic system following myelosuppressive stress^13^,^14^,^15^. Emerging evidence suggests that the inflammatory signals arising from BM endothelium during pan-haematopoietic injury can also modify HSC function^16^,^17^.

The nuclear factor (NF)-κB family of transcription factors serve as master regulators of the inflammatory response and have essential roles in haematopoiesis, including embryonic haematopoietic stem and progenitor cell (HSPC) emergence as well as survival and differentiation of haematopoietic precursors^18^,^19^,^20^. Under conditions such as bacterial infections, endothelial and immune cells express inflammatory cytokines that activate HSPCs in the BM niche^21^,^22^. Many of these cytokines are induced by canonical NF-κB signalling, including interleukin (IL)6, tumour-necrosis factor (TNF)α, interferon (IFN)γ, transforming growth factor (TGF)β, and macrophage colony-stimulating factor (M-CSF), and can regulate the proliferation and differentiation of HSPCs^23^,^24^,^25^,^26^,^27^. These signals enable the robust production of immune cells essential to counter and resolve infection. However, sustained inflammatory signalling has been shown to be detrimental to long-term HSC maintenance, resulting in a decline in their number and quality, HSC exhaustion and the emergence of haematopoietic neoplasms^28^,^29^,^30^. Based upon the physical proximity of HSCs and endothelial cells in the BM microenvironment, paracrine inflammatory signals derived from endothelial cells have been presumed to influence HSC function^29^,^30^. Endothelial cells are continuously exposed to endogenously produce inflammatory signals, such as advanced glycation end products and products of extracellular matrix breakdown like hyaluronate^31^. These advanced glycation end products and extracellular matrix components engage toll-like receptor 4 resulting in secretion of pro-inflammatory cytokines such as TNFα and IL6 that activate NF-κB signalling. Following insult to the BM microenvironment, endothelial cells produce IL1, resulting in HSC differentiation and myelopoiesis^17^. Chronic IL1 exposure has been shown to severely compromise HSC self-renewal, which is reversible upon IL1 withdrawal. In endothelial cells, NF-κB serves as a master regulator of induced expression of a vast repertoire of inflammatory cytokines^22^,^32^,^33^. Therefore, suppression of canonical NF-κB signalling specifically in endothelium could potentially rescue inflammatory signalling-mediated HSC defects^17^.

In this study, we examine the role of canonical NF-κB signalling in the maintenance and regeneration of haematopoiesis mediated by BM endothelial cells. Under steady state conditions, NF-κB inhibition augments HSC function by increasing the number of cells in quiescence and enhancing self-renewal. Moreover, the targeted inhibition of NF-κB in endothelial cells promotes rapid haematopoietic recovery following myelosuppressive injury, maintaining the integrity of the BM microenvironment, and mitigating damage to the BM endothelial cell niche. Protection of haematopoiesis and the BM vascular niche was also observed following the infusion of exogenous NF-κB-inhibited BM-derived endothelial cells (BMECs) in mice subjected to myeloablative doses of irradiation. Taken together, these results identify canonical NF-κB as a mediator of BM vascular niche function that can be modulated to enhance haematopoietic recovery following pan-haematopoietic insult.

## Results

### Endothelial NF-κB signalling modulates HSC activity *ex vivo*

**Figure 1:**
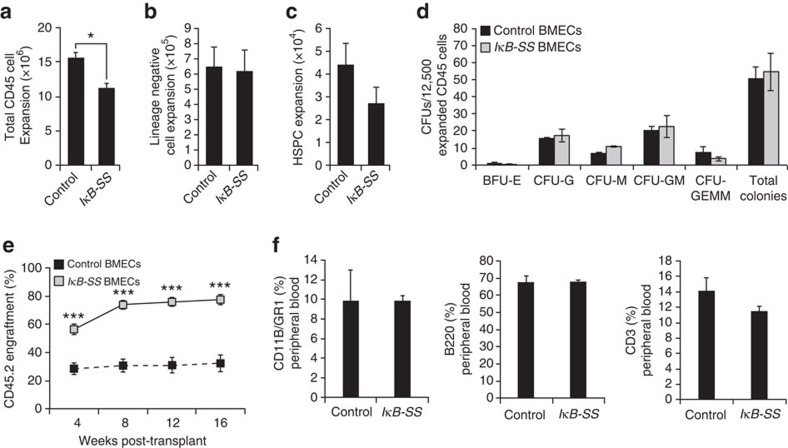
NF-κB inhibition in endothelium supports HSC activity *ex vivo*. HSPCs (cKIT^+^Lineage^−^SCA1^+^) were co-cultured *ex vivo* on control or *IκB-SS*-transduced BMECs in serum-free conditions with soluble KITL (sKITL) for nine days. (**a**–**c**) Co-cultures were analysed for (**a**) total haematopoietic cells, (**b**) lineage negative haematopoietic cells and (**c**) phenotypic HSPC expansion by flow cytometry (*n*=3). (**d**) Haematopoietic progenitor activity in BMEC-HSPC co-cultures was measured by colony-forming units (CFUs) in methylcellulose (*n*=3 per expansion). (**e**,**f**) HSC activity was quantified in a competitive repopulation assay by analysing (**e**) long-term engraftment and (**f**) multi-lineage potential in peripheral blood four months post-transplant (*n*=10 per expansion). Error bars represent mean±s.e.m. *P<0.05, **P<0.01, ***P<0.001. Pairwise comparisons were performed using Student's *t*-test; biological replicates.

To explore the possibility that NF-κB signalling in endothelial cells regulate HSPC function, we used our recently described *ex vivo* platform in which HSPCs are co-cultured on a feeder layer of murine BMECs in serum-free conditions^34^, allowing for the modulation of NF-κB signalling specifically in BMECs. To this end, we suppressed canonical NF-κB signalling in BMECs and assessed their ability to maintain and expand functional HSPCs. At rest, canonical NF-κB transcription factors are sequestered in the cytoplasm by the IκB complex. Following pathway activation, IκBα is phosphorylated at serine 32 (S32) and serine 36 (S36) by the IκB kinase (IKK), ubiquitinated, and ultimately targeted for proteasomal degradation, freeing NF-κB for translocation to the nucleus and activation of target genes via its interaction with κB binding sites in the genome^35^. To determine if constitutive inhibition of endothelial-specific canonical NF-κB signalling is sufficient to enhance the *ex vivo* expansion of repopulating HSCs, we used a dominant negative IκBα^S32A/S36A^ super suppressor (IκB-SS) construct that irreversibly binds NF-κB (p65/p50) in the cytoplasm^36^,^37^. IκB-SS protein was resistant to TNFα-mediated degradation in lentiviral-transduced *IκB-SS* BMECs (Supplementary Fig. 1a,b), inhibiting the nuclear translocation of NF-κB (p65) (Supplementary Fig. 1c,d), and functionally suppressing canonical NF-κB-mediated gene expression of cytokines known to support differentiation of HSPCs (Supplementary Fig. 1e,f) while maintaining expression of the pro-haematopoietic NOTCH ligand, JAGGED-1 (Supplementary Fig. 2a–c)^9^,^14^. To rule out NF-κB pathway crosstalk in our platform, we also examined non-canonical NF-κB signalling in *IκB-SS* BMECs. Following the stimulation of the non-canonical NF-κB pathway, NF-κB2 subunit p100 undergoes proteolytic cleavage to form p52/RelB heterodimers, activating a unique set of NF-κB genes^38^. Inhibition of canonical NF-κB in *IκB-SS* BMECs had no effect on non-canonical NF-κB signalling, ruling out *IκB-SS* transgene-mediated NF-κB crosstalk (Supplementary Fig. 1g–i). To examine whether canonical NF-κB signalling in the endothelial niche functionally alters HSPC maintenance, we utilized our *ex vivo* BMEC co-culture platform^34^. *IκB-SS*-transduced BMECs were co-cultured with cKIT^+^Lineage^−^SCA1^+^ (KLS) HSPCs derived from CD45.2 mice for nine days in serum-free conditions with exogenous soluble KITL (sKITL), and HSPC activity was assayed. *IκB-SS*-transduced BMECs were less capable of promoting total haematopoietic cell expansion (Fig. 1a), with no significant changes in phenotypic lineage negative or HSPC expansion (Fig. 1b,c). In agreement with lineage negative cell quantification, there were no observable differences in haematopoietic progenitor activity in HSPCs co-cultured on *IκB-SS*-transduced BMECs (Fig. 1d). To assess long-term repopulating activity, we transplanted 10^5^ total CD45.2^+^ co-cultured haematopoietic cells with 5 × 10^5^ CD45.1^+^ whole bone marrow (WBM) cells into lethally-irradiated CD45.1^+^ recipients and assessed multi-lineage engraftment at four months post-transplant. HSPCs cultured on *IκB-SS*-transduced BMECs displayed a dramatic increase in engraftment with no changes in their multi-lineage potential (Fig. 1e,f), demonstrating that NF-κB suppression within endothelial cells supports the *ex vivo* culture of genuine repopulating HSCs without creating a lineage-commitment bias. Conversely, pre-activation of canonical NF-κB signalling in endothelium with TNFα before expansion led to an increase in total CD45^+^ and lineage-committed haematopoietic cells, and a decrease in total phenotypic HSPCs (Supplementary Fig. 3a–e). Taken together, canonical NF-κB signalling in endothelium can modulate HSPC activity *ex vivo*, suggesting that suppressing canonical NF-κB activity in the BM vascular niche could improve the overall function of HSCs.

### Endothelial NF-κB inhibition enhances HSPC function *in vivo*

**Figure 2:**
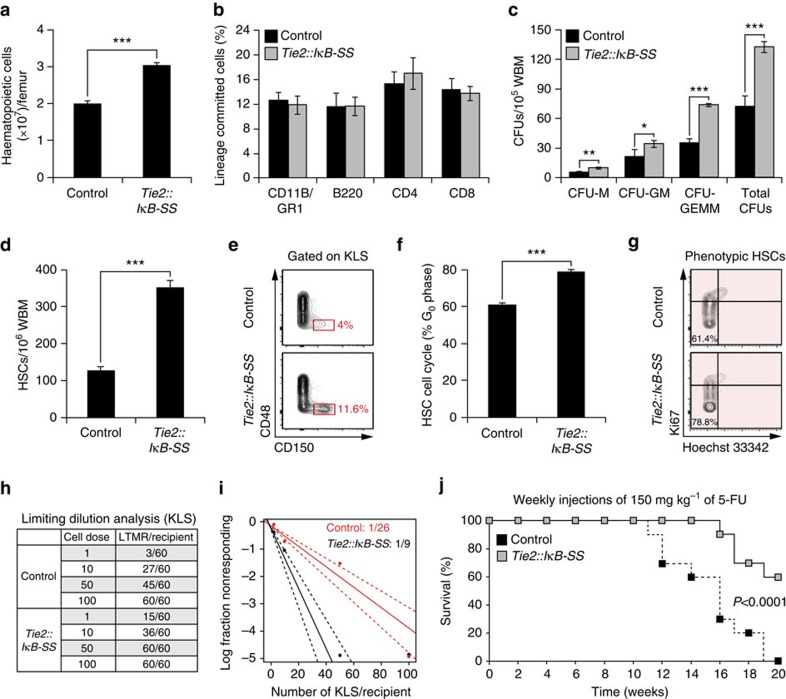
Endothelial-specific NF-κB inhibition augments homeostatic HSPC activity *in vivo*. Adult *Tie2::IκB-SS* and littermate control mice (12–16 weeks) were assayed at steady state for haematopoietic stem and progenitor cell (HSPC) activity. (**a**) Total haematopoietic cell counts per femur (*n*=5). (**b**) Percentages of lineage committed haematopoietic cells in the peripheral blood, as determined by flow cytometry (*n*=5). (**c**) Analysis of haematopoietic progenitor activity, as measured by colony-forming units (CFUs) from whole bone marrow (WBM) in methylcellulose (*n*=6). (**d**) Quantification of phenotypic haematopoietic stem cells (HSCs; cKIT^+^Lineage^−^SCA1^+^CD150^+^CD48^−^) from WBM by flow cytometry (*n*=5). (**e**) Representative contour flow plots of phenotypic HSCs from wild-type (WT) and *Tie2::IκB-SS* mice. (**f**) Quantification of cell cycle state of phenotypic HSCs. Percentages represent cells in *G*_0_ phase. (**g**) Representative cell cycle contour plots of phenotypic HSCs from WT and *Tie2::IκB-SS* mice. (**h**) Limiting dilution data demonstrating the frequency of long-term multi-lineage reconstitution of phenotypically defined HSPCs (KLS; cKIT^+^Lineage^−^SCA1^+^) (*n*=20 recipients per cell dose; biological triplicates). (**i**) A log-fraction plot of the limiting dilution analysis. Dashed lines indicate 95% confidence intervals (*n*=20 recipients per cell dose; biological triplicates). (**j**) Survival curve following weekly administration of 5-fluorouracil (5-FU) (*n*=20). Error bars represent mean±s.e.m. *P<0.05, **P<0.01, ***P<0.001. Pairwise comparisons were performed using Student's *t*-test; biological replicates.

To assess the role of NF-κB signalling within the *in vivo* BM endothelial niche, we employed a previously described transgenic mouse model in which NF-κB signalling is inhibited specifically in endothelial cells by expressing the *IκB-SS* dominant negative construct under the transcriptional control of the *Tie2* promoter (*Tie2::IκB-SS*)^39^. Under steady state conditions, *Tie2::IκB-SS* mice demonstrated a significant increase in total haematopoietic cells per femur (Fig. 2a), while maintaining similar ratios of myeloid and lymphoid cells in the peripheral blood (Fig. 2b). To examine the possibility that an increase in BM cellularity was associated with an increase in HSPC activity, we tested homeostatic HSPC function. WBM derived from wild-type (WT) control and *Tie2::IκB-SS* mice were cultured in semi-solid methylcellulose and scored for colony-forming units (CFU). *Tie2::IκB-SS* mice displayed a significant increase in CFU-M, CFU-GM, CFU-GEMM and overall CFU activity (Fig. 2c). In addition to an increase in the overall haematopoietic cell content and progenitor activity, *Tie2::IκB-SS* mice displayed a greater than threefold increase in the frequency of phenotypic HSCs (cKIT^+^Lineage^−^SCA1^+^CD150^+^CD48^−^) (Fig. 2d,e), of which significantly more *Tie2::IκB-SS* HSCs were in *G*_0_ quiescence (Fig. 2f,g). HSCs in quiescence are associated with an increase in long-term engraftment potential^40^; to this end, we tested the reconstitution ability of HSCs from *Tie2::IκB-SS* mice by performing a limiting dilution transplantation of KLS cells and assessed their long-term engraftment. We found a threefold increase in the frequency of long-term, multi-lineage repopulating cells in *Tie2::IκB-SS* mice (1/26 WT versus 1/9 *Tie2::IκB-SS*) (Fig. 2h,i), demonstrating that the observed expansion of phenotypic HSCs in *Tie2::IκB-SS* mice is associated with an increase in the frequency of *bona fide* long-term, multi-lineage repopulating HSCs. The increased frequency of HSCs and overall increase in quiescence in *Tie2::IκB-SS* mice suggests that under regenerative stress, inhibiting NF-κB signalling in endothelium may reduce the rate of premature exhaustion of HSCs. To address this, control and *Tie2::IκB-SS* mice were administered low doses of the myelosuppressive agent 5-fluorouracil (5-FU; 150 mg kg^−1^) weekly to induce cycling of HSCs and serially exhaust the HSC pool. Survival was monitored for each cohort, with all control mice succumbing to haematopoietic failure by week 19. In contrast, haematopoietic failure was delayed in *Tie2::IκB-SS* mice as evidenced by their 60% survival at week 20 (Fig. 2j), suggesting that endothelial-specific inhibition of NF-κB signalling promotes the self-renewal of HSCs under regenerative conditions.

**Figure 3:**
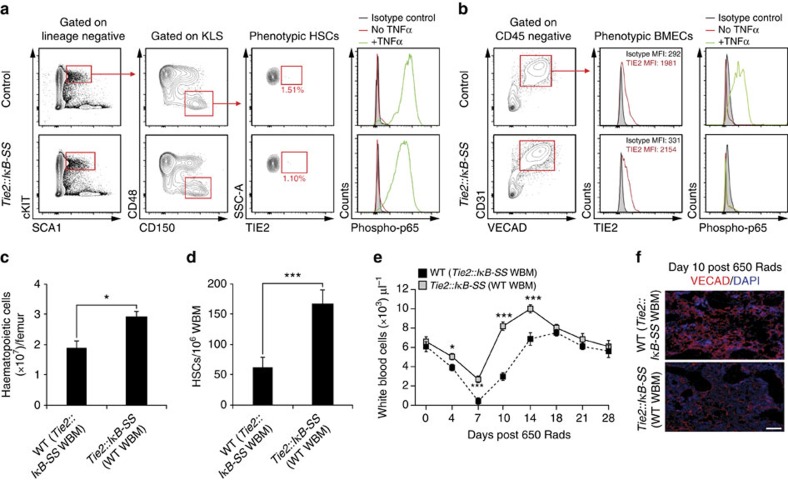
*Tie2::IκB-SS* mice do not alter NF-κB signalling in HSCs. Phenotypic haematopoietic stem cells (HSCs) and bone marrow endothelial cells (BMECs) from adult *Tie2::IκB-SS* and littermate control mice (12–16 weeks) were assessed for off-target expression. (**a**) Phenotypic HSCs (cKIT^+^Lineage^−^SCA1^+^CD150^+^CD48^−^) and (**b**) BMECs (VECAD^+^CD31^+^CD45^−^) from control and *Tie2::IκB-SS* mice were assessed for TIE2 expression and NF-κB signalling capacity (p65; phosphorylated Ser536) by flow cytometry. To assess HSC functionality in *Tie2::IκB-SS* derived WBM, reciprocal transplantations (*Tie2::IκB-SS* WBM into wild-type (WT) recipients or WT WBM into *Tie2::IκB-SS* recipients) were established and assayed four months post-transplantation. (**c**,**d**) Quantification of (**c**) total haematopoietic cell counts per femur (*n*=3) and (**d**) frequency of phenotypic HSCs following reciprocal transplantations (*n*=3). (**e**) Time course of haematopoietic recovery from inverse transplantations following myelosuppressive irradiation (*n*=7). (**f**) Representative images of intravitally labelled vasculature (VECAD) from reciprocal transplantations following myelosuppression. Scale bar, 100 μm. Error bars represent mean±s.e.m. *P<0.05, **P<0.01, ***P<0.001. Pairwise comparisons were performed using Student's *t*-test; biological replicates.

Previous reports have suggested that HSCs express *Tie2* (ref. 41). To rule out the possibility that the observed increase in HSC activity and regenerative capacity is due to HSC-specific NF-κB inhibition, we examined NF-κB activity in HSCs and BMECs. Phenotypic HSCs from control and *Tie2::IκB-SS* mice revealed diminutive levels of *Tie2* expression (Fig. 3a), and maintained their NF-κB signalling capacity following TNFα activation, as measured by phosphorylation of the NF-κB subunit p65 (Fig. 3a). TIE2^+^ BMECs isolated from *Tie2::IκB-SS* mice were unresponsive to TNFα-mediated NF-κB activation, confirming the endothelial lineage specificity of *Tie2*-driven NF-κB suppression (Fig. 3b) within the vascular niche. To further confirm that the increase in HSC activity in *Tie2::IκB-SS* mice is not due to cell autonomous inhibition of NF-κB, we transplanted equal numbers of WT control WBM into lethally irradiated *Tie2::IκB-SS* recipients and *Tie2::IκB-SS* WBM into lethally irradiated control recipients. Despite a decrease in the frequency of donor-derived HSCs from WT mice at steady state (∼127 HSCs per 10^6^ WBM; Fig. 2d), WT WBM-*Tie2::IκB-SS* recipients displayed an increase in HSC frequency (∼164 HSCs per 10^6^ WBM; Fig. 3d) and total haematopoietic cells per femur (Fig. 3c) four months post-transplant when compared with reciprocally transplanted mice. These data confirm that suppression of NF-κB signalling specifically within BM endothelium promotes HSC expansion.

### BMEC NF-κB inhibition is sufficient to augment HSC activity

The HSC supportive BM vascular niche is composed of endothelial cells and intimately associated perivascular constituents, including LEPR^+^ cells, NESTIN^+^ cells and megakaryocytes^5^. Experiments designed to delete BM niche cell-specific factors or ablate candidate cell types to explore discrete niche-HSC interactions can potentially disrupt the signalling framework within the microenvironment, resulting in unwanted changes to the numbers and function of other niche constituents. To confirm that NF-κB inhibition in BMECs is sufficient, independent of perivascular niche constituents, to promote the observed expansion of the HSC pool *in vivo*, we co-cultured WT HSPCs on BMECs derived from control or *Tie2::IκB-SS* mice under serum-free conditions with exogenous sKITL *ex vivo*. Unlike *Tie2::IκB-SS* mice at steady state (Fig. 2a,d), *ex vivo* expanded HSPCs revealed no significant differences in total CD45^+^ or phenotypic KLS populations (Supplementary Fig. 4a–c). Total expanded haematopoietic cells consisted primarily of myeloid lineages in both control and *Tie2::IκB-SS* BMEC co-cultures, with a significant increase in CD41^+^ megakaryocytes at the expense of CD11B^+^ cells (Supplementary Fig. 4d,g,h). However, similar to *in vivo Tie2::IκB-SS* mice, HSPCs co-cultured with *Tie2::IκB-SS* BMECs demonstrated more phenotypic HSPCs in G_0_ quiescence than HSPCs expanded on control BMECs (Supplementary Fig. 4e,f). To examine the long-term repopulating activity of *Tie2::IκB-SS* expanded HSPCs, we transplanted control BMEC and *Tie2::IκB-SS* BMEC-expanded CD45.2^+^ haematopoietic cells in primary and secondary competitive repopulation assays (Supplementary Fig. 4i,j). *Tie2::IκB-SS* BMEC-expanded HSPCs displayed a significant increase in long-term engraftment potential, with both control and *Tie2::IκB-SS* expansions maintaining multi-lineage potential. These data demonstrate that NF-κB inhibition in endothelial cells is sufficient to drive the observed increase in functional HSPCs and preservation of their self-renewal capabilities *in vivo*. Taken together, NF-κB signalling in the vascular niche is sufficient to modulate HSC and progenitor activity, suggesting that inhibition of NF-κB signalling in endothelial cells may be an effective therapy following myelosuppressive injury.

### NF-κB-inhibition protects the BM following myelosuppression

**Figure 4:**
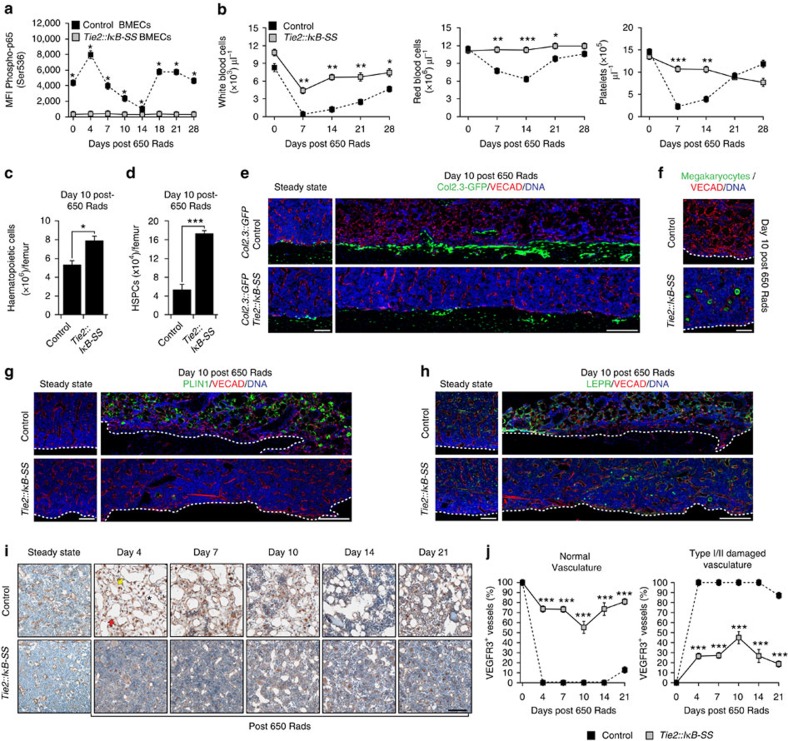
Endothelial-specific NF-κB suppression enhances haematopoietic recovery. Adult *Tie2::IκB-SS* and littermate control mice (12–16 weeks) were subjected to 650 Rads of myelosuppressive irradiation and assayed for haematopoietic recovery. (**a**) NF-κB signalling in *in vivo* BMECs (VECAD^+^CD31^+^CD45^−^) was quantified by mean fluorescence intensity (MFI) of phosphorylated p65 (Ser536) (*n*=3). (**b**) Time-course of haematopoietic recovery in peripheral blood (*n*=14, control; *n*=19, *Tie2::IκB-SS*). (**c**,**d**) Quantification of total haematopoietic cells (**c**) and phenotypic haematopoietic stem and progenitor cells (**d**) (HSPCs; cKIT^+^Lineage^−^SCA1^+^) (*n*=7, control; *n*=6, *Tie2::IκB-SS*). (**e**) Representative images of femurs from *Col2.3::GFP*; *Tie2::IκB-SS* and *Col2.3::GFP* control mice at steady state and following sublethal irradiation. Intravitally labelled vasculature (VECAD; red), *Col1a1* expressing osteoblasts (green) and nuclear staining (DAPI; blue) is noted. Scale bar, 100 μm and 200 μm, respectively. (**f**) Representative images of intravitally labelled vasculature (VECAD; red), megakaryocytes (green; citrulline) and nuclear staining (DAPI; blue). Dashed line demarcates bone. Scale bar, 100 μm. (**g**,**h**) Representative images of femurs stained with antibodies raised against PLIN1 (**g**; green) or LEPR (**h**; green), intravitally labelled vasculature (VECAD; red) and nuclear staining (DAPI; blue). Dashed line demarcates bone. Scale bar, 100 μm and 200 μm, respectively. (**i**) Representative images and (**j**) quantification of VEGFR3^+^ (brown) sinusoidal BM endothelial damage following irradiation (650 Rads). Type I haemorrhagic (asterisk), Type I discontinuous (red arrow) and Type II regressed (yellow arrow) femoral vessels are noted (*n*=3 per group). Sections were counterstained with Haematoxylin (blue). Scale bar, 200 μm. Error bars represent mean±s.e.m. (**a**) *P<0.001; (**b**–**d**,**j**) *P<0.05, **P<0.01, ***P<0.001. Pairwise comparisons were performed using Student's *t*-test; biological replicates.

To determine whether endothelial NF-κB signalling influences post-myelosuppressive haematopoietic reconstitution, we subjected *Tie2::IκB-SS* and littermate control mice to sublethal irradiation (650 Rads) and assayed for haematopoietic recovery. Following irradiation, control BMECs (CD45^−^TER119^−^VECAD^+^) demonstrated a transient spike in NF-κB signalling followed by a decrease, and ultimately returning to homeostatic levels by day 28 (Fig. 4a), which is consistent with a reported increase in WBM TNFα expression immediately following radiation-induced myelosuppression^42^. Analysis of peripheral blood in *Tie2::IκB-SS* mice revealed that endothelial-specific NF-κB inhibition protected white blood cell, red blood cell and total platelet loss following irradiation, with a rapid return to pre-irradiation levels (Fig. 4b). The observation that *Tie2::IκB-SS* mitigated peripheral haematopoietic damage following irradiation suggests that NF-κB inhibition could also protect haematopoietic tissues. To this end, we examined BM (femur) morphology in control and *Tie2::IκB-SS* mice following irradiation. Haematoxylin and Eosin (H&E) staining revealed that endothelial-specific NF-κB inhibition mitigated radiation-induced BM cytopenia at early time points (Supplementary Fig. 5; day 4), persisting throughout recovery. Because murine haematopoietic cells and BM HSPCs are highly sensitive to ionizing radiation^43^, we next examined BM haematopoietic cellularity and total HSPCs in control and *Tie2::IκB-SS* mice during active regeneration. Consistent with day 10 H&E staining (Supplementary Fig. 5), *Tie2::IκB-SS* mice displayed a significant increase in total haematopoietic cellularity and in phenotypic HSPCs per femur (Fig. 4c,d), suggesting a BM niche-mediated haematopoietic protection. To determine if endothelial NF-κB inhibition conferred a protective effect on other pro-haematopoietic niche cells within the BM microenvironment, we examined the diaphysis and trabecular regions of the BM cavity following irradiation. To this end, we crossed mice expressing *GFP* under the *Col2.3* promoter^44^ with *Tie2::IκB-SS* mice and analysed the BM microenvironment of *Col2.3::GFP*; *Tie2::IκB-SS* and *Col2.3::GFP* littermate controls at day 10 post-irradiation. Intravitally labelled endothelial cells (VECAD; red) in *Col2.3::GFP*; *Tie2::IκB-SS* mice displayed maintenance of BM integrity, including a preserved vascular network, morphology and osteoblast/osteocyte architecture (green) when compared with *Col2.3::GFP* control mice and steady state controls (Fig. 4e). Haematopoietic cells were found in tightly associated cellular packs within the BM parenchyma in *Tie2::IκB-SS* mice, whereas the BM vasculature of control mice was tortuous and devoid of dense haematopoietic cell clusters throughout the BM cavity (Fig. 4e). To further examine radiation-mediated effects to the femur morphology, we examined trabecular and cortical bone using micro computed tomography (μCT). Bone volume relative to total volume (BV/TV), trabecular and cortical bone thickness was not significantly altered in the femur of *Tie2::IκB-SS* or littermate control mice at steady state or following irradiation (Supplementary Fig. 6a,b). Interestingly, the observed recovery in white blood cells and protection of vessel morphology in *Tie2::IκB-SS* mice (Fig. 4b,e) is reflective of the *Tie2::IκB-SS*/WT WBM reciprocal transplantations following radiation-mediated myelosuppression (Fig. 3e,f), further demonstrating that the haematopoietic radio-protective effect is niche-mediated.

We next explored the possibility that the BM microenvironment is protected in *Tie2::IκB-SS* mice following irradiation. To this end, we assessed BM-niche constituents that modulate HSC activity. *Tie2::IκB-SS* BM maintained pro-haematopoietic citrulline^+^ megakaryocytes^45^ and LEPR^+^ perivascular niche cells^11^,^12^ (Fig. 4f,h), while preventing PLIN1^+^ adipocyte infiltration (Fig. 4g), which has been demonstrated to be a negative regulator of haematopoiesis^46^. BM sinusoidal endothelium undergo non-apoptotic cell death in response to ionizing irradiation^47^, while reconstitution of haematopoiesis following myelosuppressive insult is dependent on VEGFR2-mediated regeneration of VEGFR3^+^ sinusoidal endothelial cell damage *in vivo*^8^. Therefore, we set out to quantify BM VEGFR3^+^ sinusoidal endothelial regression following radiation injury. WT control mice displayed a marked increase in type I haemorrhagic/discontinuous and type II regressed femoral vessels at day 4 following injury, with noticeable sinusoidal regeneration beginning at day 14 (Fig. 4i,j). In stark contrast, *Tie2::IκB-SS* mice strongly mitigated initial damage to the femoral vessels and promoted more rapid recovery of VEGFR3^+^ sinusoids (Fig. 4i,j). Moreover, preserved sinusoidal endothelium in *Tie2::IκB-SS* mice was associated with a significant increase in circulating pro-haematopoietic granulocyte–macrophage CSF (GM-CSF) levels (Supplementary Fig. 7a,b), suggesting a combinatorial haematopoietic recovery.

To examine if the overall radio-protective effect of NF-κB inhibition in endothelium was not limited to radiation injury, *Tie2::IκB-SS* mice were administered a single myelosuppressive dose of the chemotherapeutic agent 5-FU (150 mg kg^−1^). *Tie2::IκB-SS* mice displayed a rapid recovery of total white blood cells when compared with littermate controls (Supplementary Fig. 8a,b). Unlike radiation-mediated myelosuppression, *Tie2::IκB-SS* mice displayed no distinct differences in total haematopoietic or HSPC populations within the BM at day 10 post-5-FU (Supplementary Fig. 8c,d). However, *Tie2::IκB-SS* mice did display a similar BM microenvironmental protection of vessel morphology (Supplementary Fig. 8e), preservation of citrulline^+^ megakaryocytes and LEPR^+^ niche constituents (Supplementary Fig. 8f,h), while preventing the infiltration of PLIN1^+^ adipocytes (Supplementary Fig. 8g). These data confirm that NF-κB inhibition in endothelial cells protects not only total haematopoietic cells and HSPCs in the BM following radiation injury, but also maintains the vascular niche environment. Taken together, endothelial-specific NF-κB inhibition in *Tie2::IκB-SS* mice protects the haematopoietic compartment and the integrity of the vascular niche, allowing for rapid recovery following radiation- and chemotherapeutic-mediated suppressive damage.

### *IκB-SS* BMEC infusion protects the BM niche following injury

**Figure 5:**
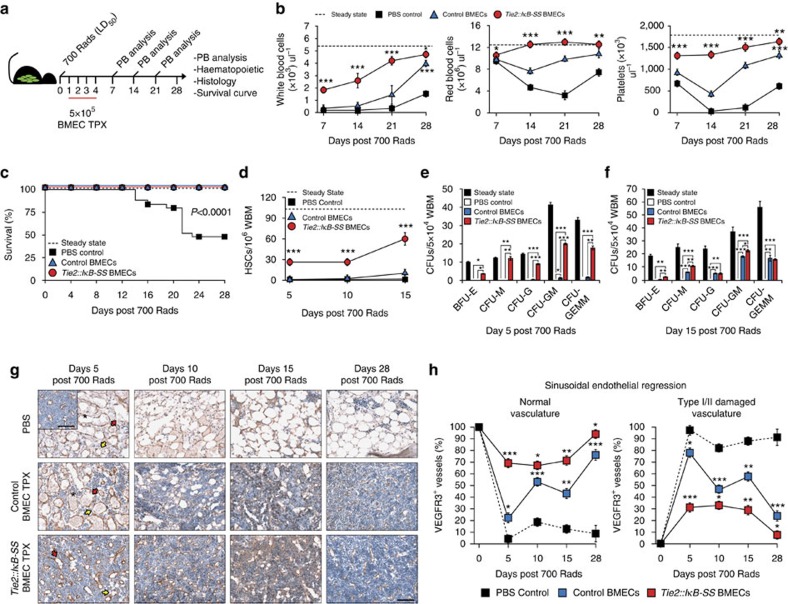
*Tie2::IκB-SS* BMEC transplantation mitigates radiation-induced myeloablation and BM sinusoidal regression. (**a**) Schematic of control and *Tie2::IκB-SS* bone marrow endothelial cell (BMEC) transplantations. Following an LD_50_ dose of irradiation (700 Rads), wild-type (WT) mice were transplanted with control or *Tie2::IκB-SS* BMECs on four successive days and assayed for haematopoietic recovery. (**b**) Time-course of haematopoietic recovery in peripheral blood (*n*=18, steady state; *n*=24, PBS control; *n*=16 WT control BMECs; *n*=19 *Tie2::IκB-SS* BMECs). (**c**) Survival curve following BMEC transplantation. (**d**) Quantification of haematopoietic stem cell (HSC; cKIT^+^Lineage^−^SCA1^+^CD150^+^CD48^−^) frequency within whole bone marrow (WBM) (*n*=5 per condition). (**e**,**f**) Analysis of haematopoietic progenitor activity in WBM, as measured by colony forming units (CFUs) in methylcellulose at day 5 (**e**) and day 15 (**f**) post-irradiation (*n*=3 per condition). (**g**) Representative images and (**h**) quantification of VEGFR3^+^ (brown) sinusoidal BM endothelial damage following irradiation (700 Rads). Type I haemorrhagic (asterisk), Type I discontinuous (red arrow) and Type II regressed (yellow arrow) femoral vessels are noted (*n*=3 per group). Sections were counterstained with Haematoxylin (blue). Scale bar, 200 μm. Note: Pairwise comparisons (**h**) are *Tie2::IκB-SS* to WT control BMECs and WT control BMECs to PBS vehicle. Error bars represent mean±s.e.m. *P<0.05, **P<0.01, ***P<0.001. Pairwise comparisons were performed using Student's *t*-test. Survival curve probability was determined using the log-rank test; biological replicates.

Transplantation of endothelial cells have been shown to mitigate radiation-induced myeloablation, support HSPC recovery and reduce the time of peripheral haematopoietic recovery *in lieu* of a haematopoietic stem cell transplantation (HSCT)^34^,^48^,^49^,^50^. Based on the data demonstrating that endogenous inhibition of endothelial NF-κB signalling augments homeostatic and regenerative haematopoiesis *in vivo*, we next sought to determine if transplantation of NF-κB-inhibited BMECs can be utilized as a cellular therapy to treat haematological disorders that require radiation and/or chemotherapy to induce remission. To this end, C57BL/6J recipients were injected with 5 × 10^5^ cultured WT control or *Tie2::IκB-SS* BMECs via retro-orbital sinus on four successive days and assayed for haematopoietic recovery following an LD_50_ dose of radiation (Fig. 5a). Although both control and *Tie2::IκB-SS* BMECs transplantations mitigated radiation-induced death (Fig. 5c), *Tie2::IκB-SS* BMECs resulted in significant enhancement of peripheral white blood cells, red blood cells and platelets when compared with control BMECs and PBS injected controls (Fig. 5b). While control BMECs also promoted significant haematopoietic recovery when compared with PBS injected controls, NF-κB inhibition further augmented the BMEC-mediated recovery with peripheral blood counts rapidly normalizing to steady state controls (Fig. 5b). We next sought to determine if NF-κB inhibition provided additional HSPC radioprotection over BMEC controls. Transplanted *Tie2::IκB-SS* BMECs preserved phenotypic HSCs at early time points following irradiation and promoted accelerated HSC recovery (Fig. 5d and Supplementary Fig. 9a,b). Accelerated recovery of phenotypic HSCs was associated with an increase in haematopoietic progenitor activity at day 5 post-irradiation, normalizing by day 15 (Fig. 5e,f). The observed increase in HSPC activity is reflective of early haematopoietic recovery noted in the peripheral blood (Fig. 5b).

Similar to the observed haematopoietic recovery following myelosuppression in *Tie2::IκB-SS* mice (Fig. 4b–d), *Tie2::IκB-SS* BMEC transplanted mice demonstrate an acceleration in peripheral blood recovery and mitigation of phenotypic and functional HSPC attrition when compared with WT BMEC transplantation or PBS injected controls (Fig. 5b–f). This raises the possibility that *Tie2::IκB-SS* BMEC transplantation following irradiation also protects the vascular niche by attenuating VEGFR3^+^ sinusoidal endothelial regression. To test this possibility, we quantified VEGFR3^+^ BM sinusoidal endothelial regression following BMEC transplantation. Mice transplanted with WT BMECs demonstrated a modest, but significant decrease in Type I and Type II damaged BM sinusoidal vessels at day 5 post-irradiation when compared with PBS vehicle controls, displaying a rapid haematopoietic and VEGFR3^+^ sinusoidal recovery by day 10 (Fig. 5g,h). These data are consistent with the pan-haematopoietic recovery previously described following exogenous BMEC transplantation^34^ (Fig. 5b–f). However, mice transplanted with *Tie2::IκB-SS* BMECs displayed a marked protection of WBM cellularity and VEGFR3^+^ sinusoidal vasculature as early as day 5 post-irradiation, when compared with WT BMEC controls (Fig. 5g,h). The mitigation of BM endothelial regression in *Tie2::IκB-SS* BMEC transplanted mice was coupled with the rapid regeneration of any residual vascular damage (Fig. 5g,h). Taken together, the intravenous delivery of exogenous *Tie2::IκB-SS* BMECs following radiation-mediated myeloablation in WT recipients protects the vascular niche in a similar manner as endogenous NF-κB inhibition, allowing for the possibility of transplanting tailored niche-specific endothelium as a therapeutic modality in treatments that affect the vascular niche.

### *IκB-SS* BMEC infusion mitigates haematopoietic damage

**Figure 6:**
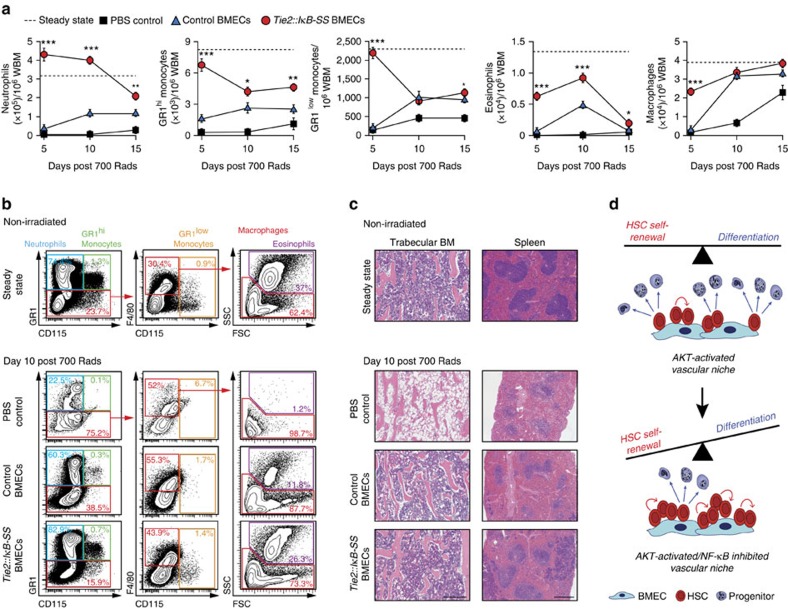
Transplantation of *Tie2::IκB-SS* BMECs safeguard haematopoietic tissues. (**a**) Quantification of the granulocyte-macrophage (GM) component of whole bone marrow (WBM) following myeloablative irradiation and control or *Tie2::IκB-SS* bone marrow endothelial cell (BMEC) transplantations (*n*=10, steady state; *n*=5, PBS control; *n*=5, control BMECs; *n*=5, *Tie2::IκB-SS* BMECs). (**b**) Representative GM contour flow plots. (**c**) Representative haematoxylin and eosin (H&E) stained sections of the bone marrow (BM) and spleen. Scale bar, 500 μm. (**d**) Model of haematopoietic stem cell (HSC) regulation by bone marrow endothelial cells (BMECs) in the vascular niche. Error bars represent mean±s.e.m. *P<0.05, **P<0.01, ***P<0.001. Pairwise comparisons were performed using Student's *t*-test; biological replicates.

Based on these observations, we hypothesized that transplantation of *Tie2::IκB-SS* BMECs provides radioprotection of haematopoietic tissues, allowing for accelerated haematopoietic recovery. To this end, we quantified reconstitution of leukocytes in WBM, as this is a critical determinant of patient outcome through the ability of leukocytes, particularly neutrophils to protect BM transplant recipients from opportunistic infections^51^,^52^,^53^. Mice transplanted with *Tie2::IκB-SS* BMECs displayed significantly higher frequencies of neutrophils, monocytes, eosinophils and macrophages at day 5 post-myeloablative irradiation (700 Rads), when compared with WT BMEC and PBS injected controls (Fig. 6a). Of note, neutrophil protection/recovery was distinct in the *Tie2::IκB-SS* BMEC transplanted mice, with the frequency of neutrophils being higher at days 5 and 10 post irradiation when compared with non-irradiated steady state controls (Fig. 6a,b). Histology of the trabecular region of the femur 10 days following irradiation in *Tie2::IκB-SS* BMEC transplants demonstrated a significant radioprotection in terms of preserved marrow and splenic cellularity when compared with WT BMECs and PBS injected control mice (Fig. 6c). *Tie2::IκB-SS* BMEC transplantations also displayed a significant protective effect on the morphology of the spleen following insult, which displayed normal architecture, including localized red and white pulp (Fig. 6c). Taken together, these data demonstrate that transplantation of exogenous *Tie2::IκB-SS* BMECs results in a profound radio-protective effect that can mitigate deleterious consequences associated with total body irradiation and accelerate recovery of peripheral haematopoietic cells.

## Discussion

The NF-κB pathway orchestrates a diverse set of biological functions, including cellular survival, proliferation and differentiation, while the dysregulation of NF-κB signalling has been implicated in the development of chronic inflammation, metabolic disorders and emerging neoplasms. These properties have made the NF-κB pathway an attractive therapeutic target for haematopoietic disorders, including the treatment of cancer and autoimmune disease^54^,^55^,^56^,^57^. In endothelial cells, the activation of pro-inflammatory NF-κB signalling in response to viral and bacterial infections can drive HSPC proliferation and differentiation in order to supplement the cellular immune response^21^,^22^. However, chronic inflammatory signalling has been shown to result in impairment of HSC repopulation potential^17^,^27^,^30^, while chronic activation of NF-κB signalling in the BM vascular niche can promote a myeloproliferative-like disorder through the expression of pro-inflammatory cytokines^30^. In addition, pre-conditioning regimens for HSCT to treat haematological malignancies and autoimmune disorders can cause systemic endothelial damage and have been implicated in the progression of HSCT-related life-threatening complications through endothelial apoptosis and pro-inflammatory cytokine production^58^,^59^. Therefore, strategies aimed at limiting pro-inflammatory responses that effect HSC maintenance and BM endothelial niche integrity may be an attractive option to preserve long-term HSC function following haematopoietic injury.

In this study, we identify the canonical NF-κB pathway as an extrinsic mediator of HSC function within the adult BM endothelial niche. Endothelial-specific inhibition of canonical NF-κB signalling using a dominant negative IκB-SS construct expressed under *Tie2* promoter elements has identified NF-κB as a key regulator within BM endothelial cells that is capable of modulating the size, cell cycle status and functionality of the HSC pool during homeostasis (Fig. 6d). NF-κB-specific inhibition in BM endothelium also promotes rapid haematopoietic recovery following chemo- and radiation-induced myelosuppression. Moreover, *Tie2::IκB-SS* mice safeguard the pro-haematopoietic BM microenvironment by preventing VEGFR3^+^ sinusoidal endothelial cell regression and suppressing the infiltration of adipocytes, negative regulators of haematopoiesis, following injury. Preservation of the sinusoidal BM vascular niche in *Tie2::IκB-SS* mice suggests that the activation of canonical NF-κB signalling following ionizing radiation promotes endothelial apoptosis *in vivo*. Indeed, numerous reports have identified endothelial cells as susceptible to irradiation- and TNFα-mediated cell death, establishing NF-κB signalling as a potential axis regulating endothelial survival following radiation-mediated myeloablation^60^,^61^. TNFα is rapidly and transiently expressed in the BM microenvironment following total body irradiation and promotes WBM cell apoptosis. Accordingly, TNFα knockout mice display a decrease in haematopoietic cell death and an increase in BM cellularity following acute radiation^42^. Considering the BM vascular niche is essential for the reconstitution of haematopoiesis following chemotherapeutic and radiation-induced insult, preserving the integrity of the BM microenvironment, particularly the endothelial niche, may provide a therapeutic opportunity to augment current HSCT approaches to increase haematopoietic engraftment and promote more rapid recovery of haematopoietic tissues following pre-conditioning regimens.

Because NF-κB signalling has been shown to promote the expression of pro-inflammatory cytokines, we analysed circulating cytokine concentrations in *Tie2::IκB-SS* mice following radiation-induced myelosuppression, identifying significant changes in M-CSF, IL6 and GM-CSF^62^,^63^,^64^ (Supplementary Fig. 7b). The observed decrease in plasma concentrations of M-CSF and IL6 coincide with the suppression NF-κB signalling in endothelium following irradiation. M-CSF and IL6 can directly promote HSPC myeloid differentiation^26^,^27^; therefore, it is plausible that the systemic downregulation of these cytokines preserves HSC functionality at the expense of differentiation. Interestingly, GM-CSF plasma levels are increased in *Tie2::IκB-SS* mice following radiation (Supplementary Fig. 7b). The administration of GM-CSF following chemotherapy in patients has been shown to increase survival, reducing the severity of neutropenia and treatment-related infections, and enhance overall haematopoietic recovery following HSCT^65^. Because GM-CSF gene expression can be induced by canonical NF-κB, the observed increase in plasma GM-CSF levels may be an indirect effect of NF-κB inhibition in endothelium. Nonetheless, increased GM-CSF levels may be at least partially responsible for promoting rapid haematopoietic regeneration following total body irradiation in *Tie2::IκB-SS* mice.

In this manuscript, we modulate NF-κB function within the BM microenvironment *in vivo* utilizing a *Tie2* promoter-driven *IκB-SS* transgenic mouse model that specifically inhibits canonical NF-κB activation in endothelial cells^39^. However, endogenous *Tie2* expression has been reported on phenotypic side population cells (HSCs), in association with its ligand Angiopoietin-1 in the BM osteoblastic niche^41^. Therefore, confirming the lineage-restricted expression of the dominant-negative *IκB-SS* transgene to the microenvironment in this report is paramount to our conclusions. Common *Tie2* core promoter/enhancer elements used to faithfully drive endothelial-specific transgene expression in multiple murine models have demonstrated that NF-κB inhibition and *cre*-mediated deletion of target genes is limited to endothelial cells^30^,^32^,^66^,^67^. Nonetheless, to rule out off-target expression of the *Tie2::IκB-SS* transgene, we examined both TIE2 expression and NF-κB functionality in phenotypic HSCs. Using the same TIE2 antibody clone described in Arai *et al*.^41^ cKIT^+^Lineage^−^SCA1^+^CD150^+^CD48^−^ HSCs from both *Tie2::IκB-SS* and littermate control mice displayed scant TIE2 staining by flow cytometry and intact canonical NF-κB activity in response to TNFα-stimulation, while freshly isolated TIE2^+^VECAD^+^CD31^+^CD45^−^ BM endothelium from *Tie2::IκB-SS* mice were functionally unresponsive to TNFα (Fig. 3a,b). Cell-autonomous canonical NF-κB signalling is critical for HSC maintenance; IκBα-dependent RelA/p65 knockout mice using either haematopoietic specific *Vav1-cre* or *Mx1-cre* in BM niche-independent reciprocal transplants demonstrated a twofold decrease in phenotypic cKIT^+^Lineage^−^SCA1^+^CD150^+^CD48^−^ HSCs, a 17-fold decrease in competitive repopulating cells, and an increase in HSC cell cycling^68^, in contrast to the increase in quiescent, long-term repopulating HSCs in *Tie2::IκB-SS* mice (Fig. 2d–i). These data verify the functional absence of IκB-SS-mediated NF-κB inhibition in *Tie2::IκB-SS* HSCs. Moreover, WT-derived WBM transplanted into irradiated *Tie2::IκB-SS* recipients displayed an increase in haematopoietic cellularity and HSC frequency in the femur at four months following reconstitution (Fig. 3c,d) reflective of steady state *Tie2::IκB-SS* mice (Fig. 2a,d), confirming a microenvironmental role of NF-κB inhibition. Myelosuppressive total body irradiation of established reciprocal transplanted mice also demonstrated a significant increase in the recovery of peripheral white blood cells and protection of the vasculature (Fig. 3e,f), similar to endogenous recovery in *Tie2::IκB-SS* mice (Fig. 4b,e). To further rule out *Tie2::IκB-SS* expression in the HSC compartment, we conducted experiments using WT HSCs. WT-derived HSCs used in our *ex vivo* BMEC co-culture platform and endogenous WT HSCs in our *in vivo* BMEC transplantation assay demonstrate the preferential expansion of long-term repopulating HSCs and protection of the BM vascular niche and haematopoietic tissues following radiation-mediated myeloablation, respectively. These results clearly demonstrate that the observed niche-mediated HSC phenotype is due to endothelial-specific NF-κB inhibition (Fig. 1, Supplementary Figs 4–6). Taken together, the inhibition of endothelial-specific NF-κB signalling in *Tie2::IκB-SS* mice promotes an increase in long-term repopulating HSCs during homeostasis, while mitigating pan-haematopoietic damage in response to chemo- and radiation-induced myelosuppression. The observed enhanced haematopoietic recovery is accompanied by preservation of the BM microenvironment, including the protection of vascular niche, and an increase in circulating GM-CSF.

The use of our *ex vivo* BMEC expansion platform to identify NF-κB signalling as a key regulatory pathway in the BM vascular niche has unlocked the potential for a cellular therapy approach to treat haematopoietic disease requiring cytotoxic regimens. Given that a delay in neutrophil and platelet engraftment is a major cause of morbidity/mortality and prolonged hospitalization following cord blood HSC transplantations^51^,^52^,^53^, the observation that *Tie2::IκB-SS* BMEC transplantation recipients maintain neutrophil counts and comparable platelet levels following an LD_50_ myeloablative dose of radiation, suggests that the co-infusion of genetically modified vascular cells could be utilized as an adjuvant cellular therapy in conjunction with HSC transplants to enhance the engraftment of HSPCs, as well as decrease the morbidity and mortality associated with radiation/chemotherapy induced pancytopenias.

## Methods

### Animal use

All animal experiments were performed under the approval of Weill Cornell Medical College and the Institutional Animal Care and Use Committee. C57BL/6J (CD45.2), B6.SJL-*Ptprc*^*a*^*Pepc*^*b*^/BoyJ (CD45.1) and B6.Cg-Tg(Col1a1*2.3-GFP)Rowe/J (*Col2.3::GFP*) mice were purchased from The Jackson Laboratory. BAC transgenic *Tie2::IκB-SS* mice were obtained from Jan Kitajewski^39^ at Columbia University and maintained on a C57BL/6J (CD45.2) background. All experiments were conducted using *Tie2::IκB-SS* and *Col2.3::GFP* hemizygous mice with the appropriate littermate negative controls. Mice were maintained in specific-pathogen-free housing and used at 12–16 weeks of age for all experiments. *Tie2::IκB-SS* and WT littermate control mice were not segregated based on sex for described experiments. For haematopoietic recovery, reconstitution and BMEC transplantation studies, mice were subjected to total body γ-irradiation (^*137*^*Cs*) 24 h before transplantation at indicated doses. Transplant recipients were given PicoLab Mouse 20 antibiotic feed (0.025% Trimethoprim and 0.124% Sulfamethoxazole; LabDiet) 24 h before irradiation until 28 days post-transplant.

### Lentivirus

The *IκB-SS* transgene was amplified using PCR Extender System (5Prime) from the pBabe-GFP-IκBα-mut vector^36^ (AddGene; plasmid#15264) with forward 5′-aggatccgccaccatgttccaggcggccgag-3′ and reverse primers 5′-agtcgactcataacgtcagacgctggcct-3′ containing BamHI and SalI sites and cloned into the Topo pCR2.1 vector (Life Technologies). The resulting pCR2.1-*IκB-SS* vector was digested with BamHI and SalI (New England Biolabs) and subcloned into the pCCL vector^69^. pCCL-*myrAkt*^13^ and pCCL-*IκB-SS* virus was generated by transfecting 13 μg of lentiviral backbone with 5 μg RRE, 2.5 μg REV and 3 μg VSV packaging plasmids into a 10 cm plate of 80% confluent 293T/17 cells (American Type Culture Collection) using Lipofectamine 2000 (Life Technologies) according to the manufacturer's suggestions. Supernatants were collected 48 h post-transfection, concentrated using Lenti-X Concentrator (ClonTech) according to the manufacturer's recommendations, resuspended in 0.5 ml TNE Buffer (50 mM Tris, pH 8.0, 1 mM EDTA, 130 mM NaCl and stored at −80 °C. Viral titer was determined using Lenti-X p24 Rapid Titer Kit (ClonTech). All transductions were done using approximately 10,000 pg virus per 30,000 BMECs cm^−1^.

### BMEC isolation

Primary murine BMECs were isolated from *Tie2::IκB-SS* or WT littermates and transduced with a constitutively-active myristoylated *Akt1*-expressing lentivirus to promote survival in serum- and cytokine-free conditions. To generate BMEC cultures, femur, tibia and humerus bones were isolated, denuded of excess tissue, crushed using a mortar and pestle and enzymatically disassociated in Hanks Balanced Salt Solution (Life Technologies) containing 20 mM HEPES (CellGro), 2.5 mg ml^−1^ Collagenase A (Roche), and 1 Unit ml^−1^ Dispase II (Roche) for 15 min at 37 °C with gentle agitation. Resulting cell suspensions were passed through a 40 μm filter, washed in PBS (pH 7.2) containing 0.5% BSA (Fraction V) and 2 mM EDTA, and depleted of terminally differentiated haematopoietic cells using a Lineage Cell Depletion Kit (Miltenyi Biotec) according to the manufacturer's suggestions. BMECs were immunopurified from resulting cell suspensions using Dynabeads (Life Technologies) pre-captured with a monoclonal CD31 antibody (MEC13.3; Biolegend). In short, 10 μl of sheep anti-rat IgG Dynabeads were incubated with 1 μg of CD31 antibody (per isolation) for 30 min at 4 °C, washed three times, added to lineage-depleted WBM in a final volume of 1 ml, and incubated for 30 min at 4 °C. Resulting CD31^+^ cells were plated and grown on fibronectin-coated (Sigma-Aldrich) plates. Antibody capture, cell isolation, and washes were done in PBS (pH 7.2) containing 0.5% BSA (Fraction V) and 2 mM EDTA. Dynabead-CD31 capture, BMEC isolations, and washes were done using a DynaMag-2 magnet (Life Technologies). C57BL/6J and *Tie2::IκB-SS* BMECs were cultured in low glucose DMEM (Life Technologies) and Ham's F-12 (CellGro) (1:1 ratio), supplemented with 20% heat-inactivated FBS, antibiotic-antimycotic (CellGro), non-essential amino acids (CellGro), 10 mM HEPES (CellGro), 100 μg ml^−1^ heparin (Sigma-Aldrich) and 50 μg ml^−1^ endothelial mitogen (Biomedical Technologies, Inc). To select for *Akt1*-expressing BMECs, cultures were starved for seven days in serum- and cytokine-free StemSpan SFEM (StemCell Technologies, Inc.) media. All BMEC lines were subsequently sorted for VECAD^+^ (BV13; Biolegend), CD31^+^ (390; Biolegend), CD45^−^ (30-F11; Biolegend) populations to ensure purity. To generate *IκB-SS* expressing endothelial cells from WT C57BL/6J endothelium, *Akt1-*BMECs were transduced with *IκB-SS* expressing lentivirus. All experiments were done using heterogeneous *pCCL-myrAkt*/*pCCL-IκB-SS*-transduced BMEC lines. Cells were cultured in humidified incubators at 37 °C under 5% CO_2_.

### *IκB-SS*-BMEC functional analysis

To confirm *IκB-SS* transduction efficiency and NF-κB pathway activation, *IκB-SS*-BMECs were serum-starved overnight in X-Vivo 20 (Lonza) media, activated by the addition of recombinant murine TNFα (PeproTech) at 20 ng ml^−1^ for 15 min, fixed/permeabilized, and stained using the following antibodies and dilutions: IκBα at 1:400 (L35A5; Cell Signaling) and secondary goat anti-rabbit Alexa Fluor 488 at 1:200 (Life Technologies), GM-CSF at 1:100 (MP1-22E9; Biolegend), IL6 at 1:100 (MP5-20F3; Biolegend), IL3 at 1:100 (MP2-8F8; Biolegend), IFNγ at 1:100 (XMG1.2; Biolegend), CD40 at 1:100 (3/23; BD Bioscience), or CD40L at 1:100 (gp39; BD Bioscience). Non-activated (No TNFα) and non-transduced BMECs served as controls. Cells were analysed using flow cytometry.

For immunocytochemical analysis, *IκB-SS*-transduced BMECs were grown on fibronectin-coated (Sigma-Aldrich) glass chamber slides (Lab-Tek) at a density of 3 × 10^4^ cells 0.7 cm^−2^, serum-starved overnight in X-Vivo 20 (Lonza) media, and activated with the addition of recombinant murine TNFα (PeproTech) at 20 ng ml^−1^ for 15 min. Following, NF-κB activation, cells were briefly washed with ice-cold PBS (pH 7.2) and fixed in 4% paraformaldehyde (PFA) in PBS (pH 7.2) for 10 min at room temperature, washed 3 × 5 min in PBS (pH 7.2), permeabilized and blocked in 5% normal goat serum (Jackson ImmunoResearch Laboratories, Inc), 1% Triton X-100, and αCD16/32 at 1:50 (93; Biolegend) in PBS (pH 7.2) for 30 min, and stained with a p65-specific antibody at 1:100 (C22B4; Cell Signaling) in 1% BSA (Fraction V), 1% Triton X-100 in PBS (pH 7.2) for 1 h at room temperature. Sections were washed 3 × 5 min in PBS (pH 7.2) and incubated in 1% BSA (Fraction V), 1% Triton X-100 in PBS (pH 7.2) with goat anti-rat Alexa Fluor 647 at 1:250 (Life Technologies) for 30 min at room temperature. All tissues were counterstained with 4,6-diamidino-2-phenylindole (DAPI) at 1 μg ml^−1^ and mounted using Prolong Gold anti-fade solution (Life Technologies). Cells were imaged on a LSM 710 confocal microscope (Zeiss).

To determine non-canonical NF-κB activation status in *IκB-SS*-transduced BMECs, cells were serum-starved overnight in X-Vivo 20 (Lonza) media, and NF-κB activated with the addition of recombinant murine TNFα (PeproTech) at 20 ng ml^−1^ for 15 min. Following, NF-κB activation, cells were briefly washed with ice-cold PBS (pH 7.2) and lysed in RIPA buffer (150 mM NaCl, 1% IGEPAL CA-630, 0.5% deoxycholate, 0.1% sodium dodecyl sulfate (SDS), and 50 mM Tris-HCl, pH 8.0) with working concentrations of PhosStop Phosphatase Inhibitor (Roche) and Complete EDTA-free Protease Inhibitor Cocktail (Roche) for 20 min at 4 °C, sonicated, and centrifuged for 10 min at 21,000*g* at 4 °C. Supernatants were stored at −80 °C. Protein concentrations were determined using the DC Protein Assay (BioRad) and 5 μg total protein was resolved on 8% SDS–acrylamide gels and electroblotted to nitrocellulose. Transferred blots were blocked for 1 h in 5% non-fat dry milk in PBS (pH 7.2) with 0.05% IGEPAL CA-630 and incubated overnight at 4 °C in 5% non-fat dry milk in PBS (pH 7.2) with 0.05% IGEPAL CA-630 with primary antibodies raised against NF-κB2 at 1:1,000 (p100/p52) (4882; Cell Signaling), JAGGED-1 at 1:1,000 (28H8; Cell Signaling), or β-tubulin at 1:1,000 (2146; Cell Signaling). Blots were washed 3 × 10 min in PBS (pH 7.2) with 0.05% IGEPAL CA-630 at room temperature and incubated in 5% non-fat dry milk in PBS (pH 7.2) with 0.05% IGEPAL CA-630 and anti-rabbit IgG (H+L) horseradish peroxidase (Jackson ImmunoResearch Laboratories, Inc) secondary antibodies at a 1:5,000 dilution for 1 h at room temperature. Blots were washed 3 × 10 min in PBS (pH 7.2) with 0.05% IGEPAL CA-630 at room temperature and developed using Amersham ECL Prime Western Blotting Detection Reagent, according to the manufacturer's suggestions. All blots were developed using Carestream Kodak BioMax Light Film (Sigma-Aldrich). All uncropped western blots can be found in Supplementary Fig. 10.

### BMEC-HSPC co-culture

Primary murine HSPCs were co-cultured on AKT-activated or AKT-activated/NF-B-inhibited BMEC feeders in serum-free conditions with sKITL supplementation. To establish co-cultures, femurs and tibia from C57BL/6J (CD45.2^+^) mice were isolated and WBM was flushed using a 26.5 gauge needle with PBS (pH 7.2) containing 0.5% BSA (Fraction V) and 2 mM EDTA. WBM was depleted of lineage^+^ cells using a Lineage Cell Depletion Kit (Miltenyi Biotec), stained using antibodies raised against cKIT at 1:100 (2B8; Biolegend) and SCA1 at 1:100 (D7; Biolegend), and sorted for KLS populations. Resulting KLS (2,500 cells per well) were plated on a single well of a 12-well dish, pre-plated with BMECs as feeders, in StemSpan SFEM serum-free media (StemCell Technologies, Inc.) with 50 ng ml^−1^ recombinant murine sKITL (PeproTech). Note: Individual BMEC wells were washed three times with PBS (pH 7.2) before plating/splitting to remove any residual EC growth media. To test each individually isolated WT or *IκB-SS*-transduced/*Tie2::IκB-SS*-derived BMEC line (*n*=3), three independent KLS populations were used in parallel for each individual co-culture experiment. Cells were co-cultured at 37 °C and 5% CO_2_ for nine days and split as follows: (day 2) Co-cultures were supplemented with 50 ng ml^−1^ sKITL; (day 4) co-cultures were supplemented with 0.5 mls StemSpan SFEM and 50 ng ml^−1^ sKITL; (day 6) non-adherent haematopoietic cells were collected, centrifuged at 500*g* for 5 min, pellets were resuspended in 3 mls StemSpan SFEM, and 1.5 mls were plated onto the original and adjacent 12-well with BMECs (1:2 split) and supplemented with 50 ng ml^−1^ sKITL; (day 8) co-cultures were split 1:2 by transferring 0.75 ml of non-adherent haematopoietic cells onto adjacent 12-wells (total 4 × 12-wells per expansion), adding back 0.75 mls StemSpan SFEM to the original wells (1.5 mls total culture volume per 12-well), and supplementing with 50 ng ml^−1^ sKITL; (day 9) BMECs and total haematopoietic cells were collected using Accutase (Biolegend) and analysed. Total cell numbers were determined using haemocytometer counts with Trypan Blue (Life Technologies) for live/dead exclusion. Lineage^−^ haematopoietic cell composition following co-culture was assessed using the following antibodies and dilutions: GR1 at 1:100 (RB6-8C5; Biolegend), CD11B at 1:100 (M1/70; Biolegend), B220 at 1:100 (RA3-6B2; Biolegend), CD3 at 1:50 (17A2; Biolegend), CD41 at 1:100 (MWReg30; Biolegend) and CD45 at 1:100 (30-F11; Biolegend). Lineage^+^ haematopoietic cell composition following co-culture was assessed using the following antibodies and dilutions: GR1 at 1:100 (RB6-8C5; Biolegend), CD11B at 1:100 (M1/70; Biolegend), CD41 at 1:100 (MWReg30; Biolegend), and CD45 at 1:100 (30-F11; Biolegend). To assess HSC activity, total co-culture cells were stained with an antibody raised against CD45.2 at 1:100 (104; Biolegend), sorted to purity, and 10^5^ CD45.2^+^ expansion cells were transplanted into lethally-irradiated CD45.1 recipients (950 Rads) with 5 × 10^5^ CD45.1^+^ competitive WBM (per mouse) via retro-orbital injection. For secondary analysis, WBM cells were isolated from primary recipients 16 weeks post-transplantation and 10^6^ total WBM cells were transplanted into lethally-irradiated (950 Rads) CD45.1^+^ recipients (per mouse) via retro-orbital injection, as described above. Multi-lineage engraftment in peripheral blood was assayed four months-post transplant. For endothelial NF-κB pre-activation, BMECs were serum-starved overnight followed by the addition of 10 ng ml^−1^ recombinant murine TNFα for 20 min. Following NF-κB endothelial activation, wells were washed three times with PBS (pH 7.2) to remove residual TNFα and HSPCs were then plated.

### Flow cytometry and cell sorting

Before staining, F_c_ receptors were blocked with a CD16/32 antibody at 1:50 (93; Biolegend) in PBS (pH 7.2) containing 0.5% BSA (Fraction V) and 2 mM EDTA for 10 min at 4 °C. For cell surface staining, samples were stained for 30 min at 4 °C with fluorochrome-conjugated antibodies according to the manufacturer's recommendation. Stained cells were washed in blocking buffer and fixed in 1% PFA in PBS (pH 7.2) with 2 mM EDTA for flow analysis or resuspended in PBS (pH 7.2) with 2 mM EDTA and 1 μg ml^−1^ DAPI (Biolegend) for sorting. For intracellular staining, cells were processed with BD Phosflow Fix Buffer I (BD Bioscience) and BD Phosflow Perm Buffer III (BD Bioscience) according to the manufacturer's suggestions and stained with primary fluorochrome-conjugated antibodies as described. Samples were analysed using a LSR II SORP (BD Biosciences) and sorted using an ARIA II SORP (BD Biosciences). Data was collected and analysed using FACs DIVA 8.0.1 software (BD Biosciences). All gating was determined using unstained controls and fluorescence minus one strategies.

### WBM analysis

To quantify total BM haematopoietic cell counts at steady state and during recovery, femurs were isolated, denuded of excess tissue, crushed using a mortar and pestle and enzymatically disassociated in Hanks Balanced Salt Solution (Life Technologies) containing 20 mM HEPES (CellGro), 2.5 mg ml^−1^ Collagenase A (Roche), and 1 Unit ml^−1^ Dispase II (Roche) for 15 min at 37 °C with gentle agitation. Resulting cell suspensions were passed through a 40 μm filter and washed in PBS (pH 7.2) containing 0.5% BSA (Fraction V) and 2 mM EDTA. Total cell numbers were determined using haemocytometer counts with Trypan Blue (Life Technologies) for live/dead exclusion. To assess haematopoietic populations, WBM was isolated from femurs crushed with a mortar and pestle in PBS (pH 7.2) containing 0.5% BSA (Fraction V) and 2 mM EDTA, and stained for phenotypic resident HSC, HSPC and myeloid cell surface markers using the following antibodies and dilutions: HSC/HSPC; cKIT at 1:100 (2B8; Biolegend), SCA1 at 1:100 (D7; Biolegend), CD150 at 1:100 (TC15-12F12.2; Biolegend), CD48 at 1:100 (HM48-1; Biolegend) and lineage^+^ GR1 at 1:100 (RB6-8C5; Biolegend), CD11B at 1:100 (M1/70; Biolegend), B220 at 1:100 (RA3-6B2; Biolegend), CD3 at 1:50 (17A2; Biolegend), CD41 at 1:100 (MWReg30; Biolegend), TER119 at 1:100 (TER119; Biolegend), and myeloid; GR1 at 1:100 (RB6-8C5; Biolegend), CD115 at 1:100 (AFS98; Biolegend), F4/80 at 1:100 (BM8; Biolegend). Samples were analysed using flow cytometry.

### Peripheral blood

Peripheral blood was collected using 75 mm heparinized glass capillary tubes (Kimble-Chase) via retro-orbital sinus at indicated time points. To assess haematopoietic recovery following myeloablation, white blood cell, red blood cell and platelet populations were analysed using an Advia120 (Bayer Healthcare). To quantify lineage^+^ haematopoietic cells and HSC engraftment, peripheral blood was depleted of red blood cells (RBC lysis; Biolegend), stained with pre-conjugated antibodies and analysed using flow cytometry. Antibodies and dilutions used for lineage^+^ haematopoietic cell analysis: GR1 at 1:100 (RB6-8C5; Biolegend), CD11B at 1:100 (M1/70; Biolegend), B220 at 1:100 (RA3-6B2; Biolegend), CD3 at 1:50 (17A2; Biolegend), at 1:100 CD4 (GK1.5; Biolegend), and CD8 at 1:100 (53-6.7; Biolegend). Antibodies used for haematopoietic engraftment: CD45.1 at 1:100 (A20; Biolegend), CD45.2 at 1:100 (104; Biolegend), TER119 at 1:100 (TER119; Biolegend).

### Progenitor activity

Haematopoietic progenitor activity was assessed by quantifying CFUs in semi-solid methylcellulose. For *in vivo* analysis, WBM was flushed from isolated femurs using a 26.5 gauge needle with PBS (pH 7.2) containing 0.5% BSA (Fraction V) and 2 mM EDTA at indicated time points, counted using a haemocytometer with Trypan Blue (Life Technologies) for live/dead exclusion, and plated (in duplicate) in Methocult GF M3434 methylcellulose (StemCell Technologies) according to the manufacturer's protocol. For *ex vivo* expansion analysis, total haematopoietic cells were stained with anti-CD45 at 1:100 (30-F11; Biolegend), sorted, and plated as described above. Colonies were viewed using a SZX16 Stereo-Microscope (Olympus) and scored for phenotypic CFU-GEMM, CFU-GM, CFU-G, CFU-M and BFU-E colonies.

### Cell cycle

Cell cycle analysis was performed on phenotypically defined *in vivo* steady state HSCs (cKIT^+^Lineage^−^SCA1^+^CD150^+^CD48^−^) and expanded *ex vivo* HSPCs (CD45^+^cKIT^+^Lineage^−^SCA1^+^) using the following fluorescently conjugated antibodies and dilutions: cKIT at 1:100 (2B8; Biolegend), SCA1 at 1:100 (D7; Biolegend), CD150 at 1:100 (TC15-12F12.2; Biolegend), CD48 at 1:100 (HM48-1; Biolegend) and lineage^+^ GR1 at 1:100 (RB6-8C5; Biolegend), CD11B at 1:100 (M1/70; Biolegend), B220 at 1:100 (RA3-6B2; Biolegend), CD3 at 1:50 (17A2; Biolegend), CD41 at 1:100 (MWReg30; Biolegend), TER119 at 1:100 (TER119; Biolegend) and CD45 at 1:100 (30-F11; Biolegend). In short, flushed WBM or total BMEC-HSPC co-cultures were stained for HSC or HSPC surface markers, as described above. Cells were then fixed/permeabilized and stained with an antibody raised against Ki67 (16A8; Biolegend) and counterstained with Hoechst 33342 (BD Biosciences), according to the manufacturer's recommendations. Samples were analysed using flow cytometry.

### Limiting dilution

To isolate HSPCs, femurs and tibiae were harvested from *Tie2::IκB-SS* mice and littermate controls (CD45.2^+^), flushed using a 26.5 gauge needle with PBS (pH 7.2) containing 0.5% BSA (Fraction V) and 2 mM EDTA to recover WBM, and depleted of lineage^+^ haematopoietic cells using the murine Lineage Cell Depletion Kit (Miltenyi Biotec) according to the manufacturer's suggestions. Phenotypically defined HSPCs (cKIT^+^SCA1^+^) were stained and live cells (DAPI^−^) were sorted from resulting lineage-depleted WBM for competitive transplantation. For limiting dilution analysis, indicated numbers of HSPC (KLS) were transplanted into lethally irradiated CD45.1 recipients (950 Rads) with 5 × 10^5^ CD45.1^+^ competitive WBM (per mouse) via retro-orbital injection. Percent negative mice were determined six months post-transplant by assessing the contribution of CD45.2^+^ haematopoietic engraftment in the peripheral blood, as described above. Transplanted mice displaying <1% CD45.2^+^ contribution were qualified as negative engraftment. HSC frequency and 95% confidence intervals were determined using ELDA software (http://bioinf.wehi.edu.au/software/elda/)^70^.

### HSC self-renewal

*Tie2::IκB-SS* mice and littermate controls were subjected to weekly administration of the chemotherapeutic drug 5-fluorouracil (5-FU; 150 mg kg^−1^) (Sigma-Aldrich) via intraperitoneal injection. Mice were monitored daily for moribundity and survival daily.

### *In vivo Tie2::IκB-SS* expression

*Tie2::IκB-SS* and littermate control mice were intravitally labelled for 10 min with 25 μg Alexa Fluor 647-conjugated VECAD antibody (BV13; Biolegend) via retro-orbital sinus injection, mice were killed and perfused via intracardiac injection with PBS (pH 7.2). Femurs and tibiae were harvested and crushed using a mortar and pestle and enzymatically digested in Hanks Balanced Salt Solution (Life Technologies) containing 10 mM HEPES (pH 7.2) (CellGro) and 2.5 mg ml^−1^ Collagenase A (Roche) and 1 unit ml^−1^ Dispase II (Roche) for 15 min at 37 °C with gentle agitation. Subsequent cell suspensions were washed using PBS (pH 7.2) containing 0.5% BSA (Fraction V) and 2 mM EDTA and depleted of lineage^+^ haematopoietic cells using a Lineage Cell Depletion Kit (Miltenyi Biotec), according to the manufacturer's suggestions.

To assess *Tie2* expression, lineage-depleted WBM was stained for phenotypic HSC and BMEC surface markers using the following antibodies and dilutions: HSCs; cKIT at 1:100 (2B8; Biolegend), SCA1 at 1:100 (D7; Biolegend), CD150 at 1:100 (TC15-12F12.2; Biolegend), CD48 at 1:100 (HM48-1; Biolegend) and BMECs; CD31 at 1:100 (390; Biolegend). Resulting cells were washed, fixed/permeabilized and stained with an antibody raised against TIE2 at 1:20 (TEK4; Biolegend).

To assess NF-κB activation potential, lineage-depleted WBM was incubated with 20 ng ml^−1^ of murine recombinant TNFα (PeproTech) in X-Vivo 20 (Lonza) media for 20 min at 37 °C and stained for phenotypic HSC and BMEC surface markers as described above. Resulting cells were washed, fixed/permeabilized and stained with a primary antibody raised against phosphorylated p65 at 1:1,000 (93H1; Cell Signaling), and secondary antibody goat anti-rabbit Alexa Fluor 488 at 1:250 (Life Technologies), according to the manufacturer's recommendations. Isotype controls were used to establish signalling baselines. Samples were analysed using flow cytometry.

### Reciprocal transplantation

Long-bones from *Tie2::IκB-SS* or littermate control mice were isolated and WBM was flushed using a 26.5 gauge needle with PBS (pH 7.2) containing 0.5% BSA (Fraction V) and 2 mM EDTA. 10^6^ WBM cells in PBS (pH 7.2) were transplanted into lethally irradiated (950 Rads) reciprocal recipients (per mouse) and assayed 16 weeks post-transplant for total haematopoietic cells per femur, HSCs per femur and haematopoietic recovery following myelosuppression.

### *In vivo* histology and immunohistochemistry

Histologic analysis for control and *Tie2::IκB-SS* mice following 650 Rads of total body irradiation or control and *Tie2::IκB-SS* BMEC transplantation following 700 Rads of total body irradiation was performed by Histoserv, Inc (Germantown, MD). In short, killed mice were perfused with PBS (pH 7.2) via intracardiac injection, tissues were fixed in 4% PFA in PBS (pH 7.2) overnight at 4 °C, decalcified for 72 h in 10% EDTA at room temperature and dehydrated in 70% ethanol. Paraffin embedded sections (6 μm) were stained with H&E. BM and spleen images were taken on a BX51 (Olympus) light microscope at 10 × and 20 × magnification, respectively.

To analyse VEGFR3^+^ BM sinusoidal regression, paraffin embedded sections (6 μm; Histoserv, Inc) from *Tie2::IκB-SS* mice following 650 Rads of total body irradiation or control and *Tie2::IκB-SS* BMEC transplantation following 700 Rads of total body irradiation were deparaffinized in Xylene (VWR) and rehydrated through serial ethanol washes as follows: Xylene 2 × 3 min; 100% ethanol 2 × 3 min; 95% ethanol 1 × 3 min; 80% ethanol 1 × 3 min; 70% ethanol 1 × 3 min; 50% ethanol 1 × 3 min and water rinse. Antigens were retrieved by incubating rehydrated sections in 10 mM sodium citrate and 0.05% Tween-20 at 95 °C-100 °C for 20 min. Sections were quenched for peroxidase activity with 0.3% H_2_O_2_ for 30 min at room temperature, antigen blocked with 2.5% Normal Goat Serum (ImmPRESS Kit; Vector Laboratories), and incubated with either a VEGFR3 antibody (AFL4; Biolegend) at 20 μg ml^−1^ or IgG isotype control antibody (HTK888; Biolegend) at 20 μg ml^−1^ overnight at 4 °C in Normal Goat Serum, according to the manufacturer's suggestions. Stained sections were washed, incubated in ImmPRESS Reagent (Vector Laboratories), and developed using ImmPACT DAB peroxidase substrate (Vector Laboratories), according to the manufacturer's protocol. Sections were washed for 10 min in water and counterstained in Mayer's Haematoxylin Solution (Sigma) for 6 min, and cleared through serial dehydration with ethanol and xylene as follows: 70% ethanol 1 × 3 min; 95% ethanol 1 × 3 min; 100% ethanol 2 × 3 min; Xylene 2 × 3 min. Sections were mounted using DPX Mountant medium (Sigma). Images were taken on a BX51 (Olympus) light microscope at 20 × magnification.

For immunohistochemical analysis, *Tie2::IκB-SS, Col2.3-GFP* and WT control mice were intravitally labelled for 10 min with 25 μg Alexa Fluor 647-conjugated VECAD antibody (BV13; Biolegend) via retro-orbital sinus injection, mice were killed and perfused via intracardiac injection with PBS (pH 7.2). Femurs were fixed overnight with 4% PFA in PBS (pH 7.2), washed in PBS (pH 7.2) and decalcified for 72 h in 10% EDTA at room temperature. Femurs were washed in PBS (pH 7.2), cryopreserved in 30% sucrose for 48 h at 4 °C, and embedded in 50% optimal cutting temperature (OCT). (Tissue-Tek) and 50% sucrose. Sections (12 μm) were cut on a CM 3050S Cryostat (Leica). For staining, femurs were blocked for 30 min at room temperature in 10% normal goat serum (Jackson ImmunoResearch Laboratories, Inc), 0.1% Triton X-100, and αCD16/32 at 1:50 (citrulline/PLIN1) or 10% normal donkey serum (Jackson ImmunoResearch Laboratories, Inc), 0.1% Triton X-100, and αCD16/32 at 1:50 (LEPR), and stained in blocking buffer with antibodies raised against citrulline at 1:300 (EMD Millipore), PLIN1 at 1:100 (Sigma), or Alexa Fluor 594 (Life Technologies) pre-conjugated LEPR at 1:50 (R&D Systems) overnight at 4 °C. Sections were washed in PBS (pH 7.2) and citrulline/PLIN1 sections were incubated in blocking buffer with goat anti-rabbit Alexa Fluor 594 at 1:250 (Life Technologies) for 30 min at room temperature. All tissues were counterstained with DAPI at 1 μg ml^−1^ and mounted using Prolong Gold anti-fade solution (Life Technologies). Longitudinal femur sections were imaged on a LSM 710 confocal microscope (Zeiss).

### μCT analysis

A Scanco Medical μCT 35 system with an isotropic voxel size of 7 μm was used to image the distal femur. Scans used an X-ray tube potential of 55 kVp, an X-ray intensity of 0.145 mA and an integration time of 600 ms. For trabecular bone analysis, an upper 2.1-mm region beginning 280 μm proximal to the growth plate was contoured. For cortical bone analysis, a region 0.6 mm in length centered on the midshaft was used. Trabecular and cortical bones were thresholded at 211 and 350 mgHA cm^−3^, respectively. Three-dimensional images were obtained from contoured two-dimensional images by methods based on distance transformation of the binarized images. All images presented are representative of their respective experimental groups.

### Plasma cytokine analysis

Whole blood was collected via retro orbital bleeds using 150 mm heparinized glass capillary tubes (Kimble Chase) into 20 μl of 10 mM EDTA in PBS (pH 7.2) and centrifuged at 1,000*g* for 15 min at 4 °C. Resulting plasma supernatants were stored at −80 °C. Plasma was analysed using the Luminex xMAP platform (EMD Millipore) according to the manufacturer instructions. In short, plasma samples were diluted 1:2 in assay buffer and analysed using the following mouse panels: Mouse Angiogenesis/Growth Factor Magnetic Bead Panel, including TNFα, IL1β, IL6, G-CSF, SDF1α, FGF2, ANGPT2, EGF, MCP1, Endothelin-1 and Endoglin (EMD Millipore; MAGPMAG-24K) or Mouse Cytokine/Chemokine Magnetic Bead Panel, including IL3, IFNγ, GM-CSF, and M-CSF (EMD Millipore; MCYTOMAG-70K). Samples were run using the Luminex FLEXMAP 3D system and resulting data was analysed with MILLIPLEX Analyst Software.

### BMEC transplantation

C57BL/6J mice were subjected to a myeloablative dose of total body γ-irradiation (700 Rads) in which 50% of untreated mice succumb to haematopoietic failure (LD_50_; optimal dosing determined in-house). C57BL/6J irradiated recipients were transplanted with 5 × 10^5^ WT BMECs, 5 × 10^5^ *Tie2::IκB-SS* cultured BMECs or PBS (carrier) controls on four successive days via retro-orbital sinus injection following total body irradiation. Mice were monitored for survival and haematopoietic recovery at indicated time points post-irradiation.

### Statistics

No statistical models were employed to predetermine experimental sample sizes. Significance of pairwise comparisons were determined using unpaired Student's *t*-tests, with a significance threshold set at *P*<0.05. All values are presented as mean±s.e.m. Survival curve significance was calculated using the log-rank test.

### Data availability

The authors declare that the data supporting the findings within this study are available within the manuscript and its Supplementary Files and are also available from the corresponding author upon reasonable request.

## Additional information

**How to cite this article:** Poulos, M. G. *et al*. Endothelial-specific inhibition of NF-κB enhances functional haematopoiesis. *Nat. Commun.* **7,** 13829 doi: 10.1038/ncomms13829 (2016).

**Publisher's note:** Springer Nature remains neutral with regard to jurisdictional claims in published maps and institutional affiliations.

## Supplementary Material

Supplementary Information Supplementary Figures
