# Supplementary material for: Endothelial-specific inhibition of NF-κB enhances functional haematopoiesis
Source: Nat Commun. 2016 Dec 21;7:13829. doi: 10.1038/ncomms13829 (PMC5187502; doi:10.1038/ncomms13829)
Supplement: Supplementary Information — Supplementary Figures [file ncomms13829-s1.pdf]

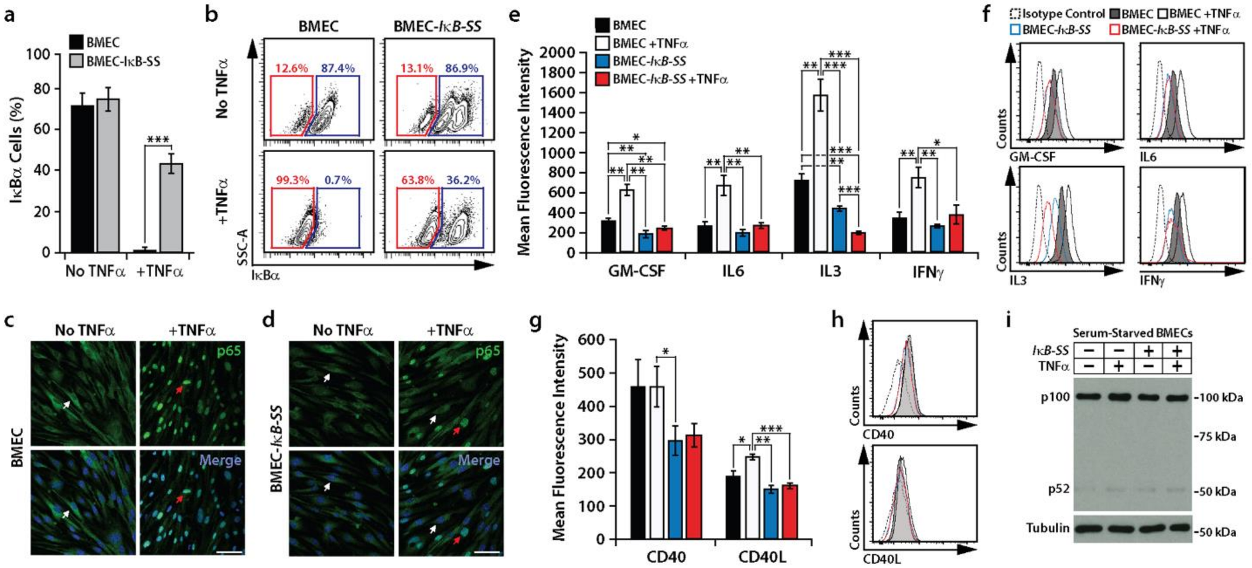

Supplementary Figure 1. IκB-SS inhibits canonical NF-κB signaling in BMECs. a, Quantification of IκBα in unstimulated and TNFα-stimulated control and *IκB-SS*-transduced BMECs, by intracellular flow cytometry (n = 3). **b,** Representative contour plots of control and *IκB-SS*-transduced BMECs following NF-κB activation. Note: Only endogenous IκBα is responsive to TNFα-mediated degradation. **c-d,** Images of **(c)** control and **(d)** *IκB-SS*-transduced BMECs stained for NF-κB subunit p65 (green) and DAPI (blue) following NF-κB activation. Cytoplasmic p65 = inactive NF-κB (white arrowhead); nuclear p65 = activated NF-κB (red arrowhead). Scale bar = 50 μm. **e,** Quantification of NF-κB-dependent gene expression in control and *IκB-SS*-transduced BMECs following TNFα stimulation, by intracellular flow cytometry (n = 3). **f,** Representative histograms of control and *IκB-SS*-transduced BMECs following NF-κB activation. **g,** Quantification of non-canonical NF-κB2 receptor (CD40) and ligand (CD40L). Note: Legend in panel (e). **h,** Representative contour plots in control and *IκB-SS*-transduced BMECs. Note: Legend in panel (f). **i,** Western blot of NF-κB2 in TNFα-activated control and *IκB-SS*-transduced BMECs. p100 = Inactive NF-κB2; p52 = activated NF-κB2. Error bars represent mean ± SEM. *P<0.05, **P<0.01, ***P<0.001. Pairwise comparisons were performed using Student's t-test; biological replicates.

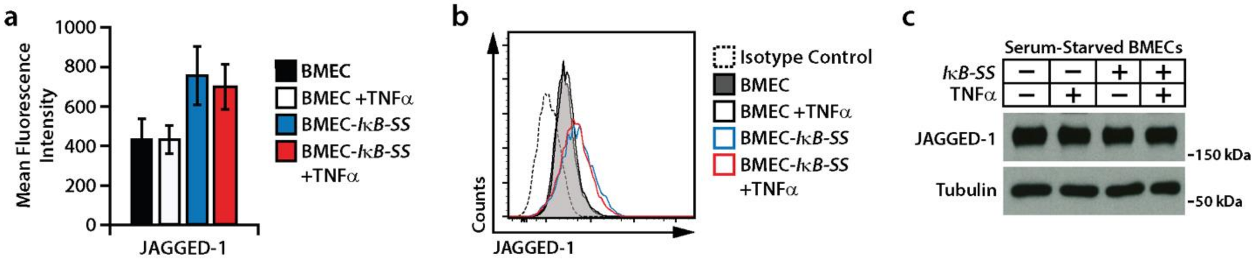

Supplementary Figure 2. IκB-SS-mediated suppression of canonical NF-κB signaling does not alter JAGGED-1 expression in BMECs. a, Quantification of JAGGED-1 expression in control and *IκB-SS*-transduced BMECs following TNFα stimulation, by flow cytometry (n = 3). Note: No significant changes were observed. **b,** Representative histograms of control and *IκB-SS*-transduced BMECs following NF-κB activation. **c,** Western blot of JAGGED-1 expression in TNFα-activated control and *IκB-SS*-transduced BMECs. Error bars represent mean ± SEM. *P<0.05, **P<0.01, ***P<0.001. Pairwise comparisons were performed using Student's t-test; biological replicates.

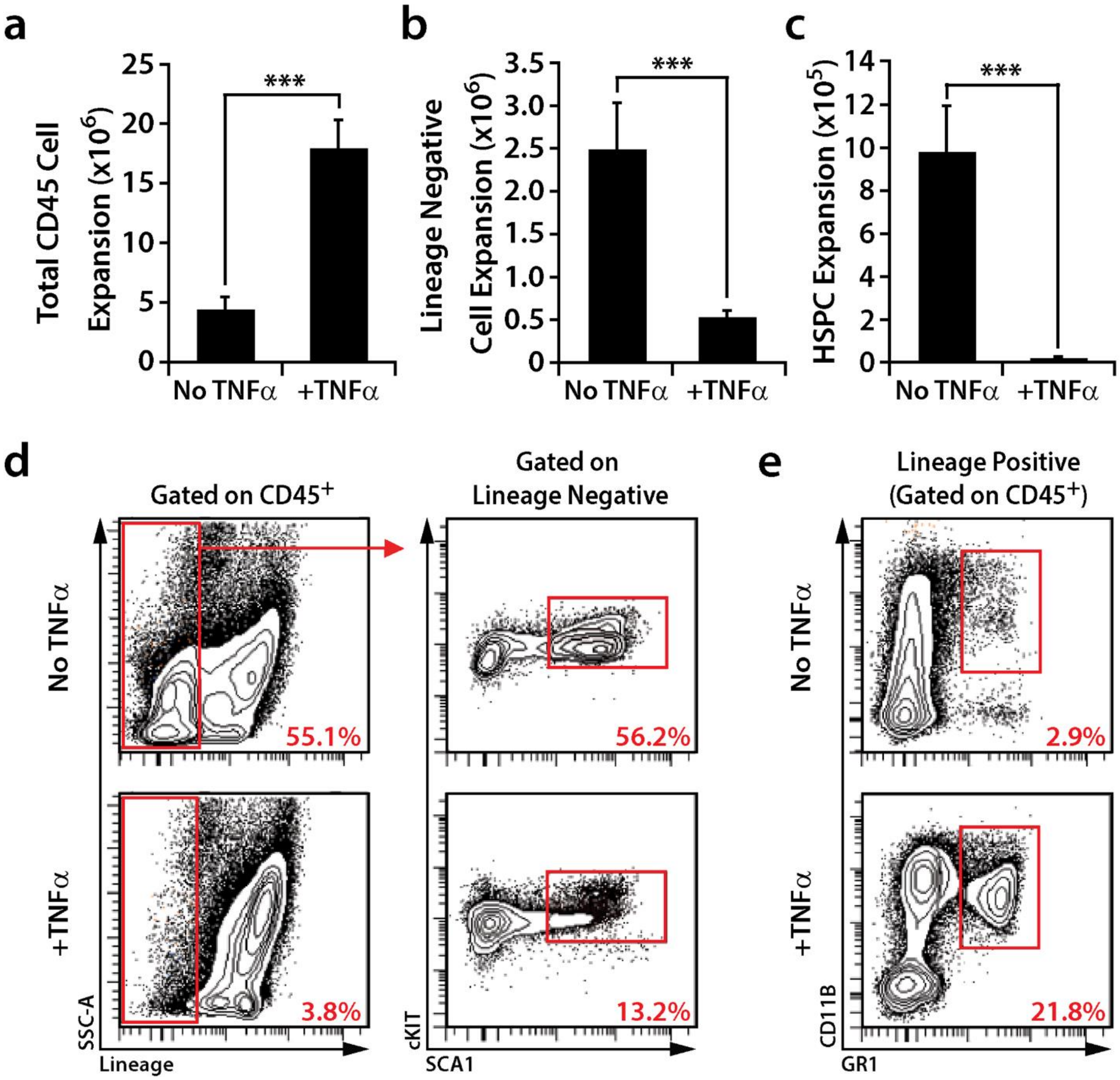

Supplementary Figure 3. Endothelial NF-κB activation inhibits phenotypic HSPC expansion *ex vivo.* **a-c,** Endothelial cells were pre-treated with TNFα and co-cultured with HSPCs in serum free-media with soluble KITL for nine days and assessed for **(a)** total hematopoietic, **(b)** lineage negative, and **(c)** HSPCs expansion by flow cytometry (n = 3). **d-e,** Representative contour plots of **(d)** HSPC and **(e)** lineage positive cells following expansion. Error bars represent mean ± SEM. *P<0.05, **P<0.01, ***P<0.001. Pairwise comparisons were performed using Student's t-test; biological replicates.

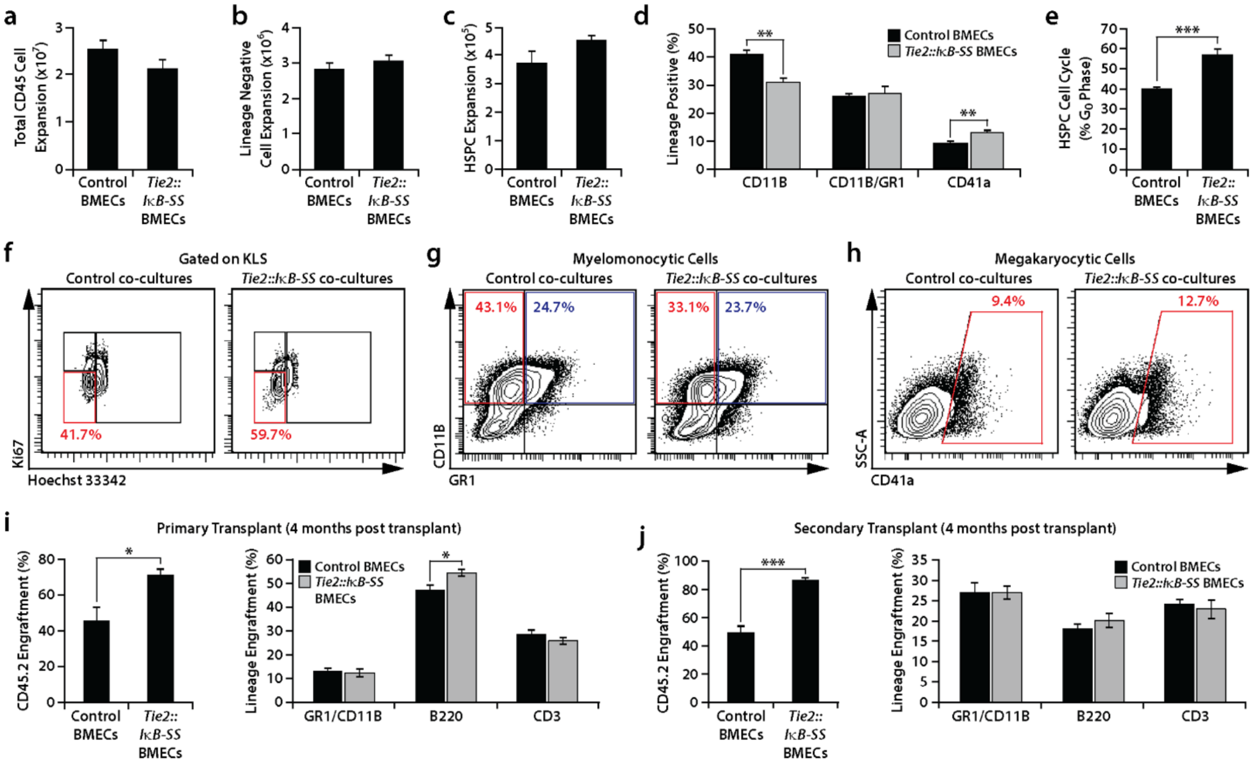

Supplementary Figure 4. ***Tie2::IκB-SS*****-derived BMECs support long-term repopulating HSCs *ex vivo*.** HSPCs were co-cultured with BMECs derived from *Tie2::IκB-SS* and littermate control mice in serum-free media with soluble KITL for nine days and assessed for phenotypic hematopoietic populations and long-term repopulating HSC activity. **a-d,** Quantification of total **(a)** hematopoietic cell, **(b)** lineage negative, and **(c)** HSPC ($cKIT^+Lineage^-SCA1^+$) expansion and **(d)** lineage positive populations by flow cytometry (n = 3). **e,** Cell cycle analysis of co-cultured HSPCs (n = 3). **f-g**, Representative contour plots of **(f)** expanded HSPC cell cycle analysis, **(g)** myelomonocytic, and **(h)** megakaryocyte cell populations. **i-j,** HSC activity was assessed by competitive repopulation and quantifying **(i)** primary and **(j)** secondary recipient engraftment and multilineage potential in peripheral blood four months post-transplant by flow cytometry (n = 10 mice/expansion). Error bars represent mean ± SEM. *P<0.05, **P<0.01, ***P<0.001. Pairwise comparisons were performed using Student's t-test; biological replicates.

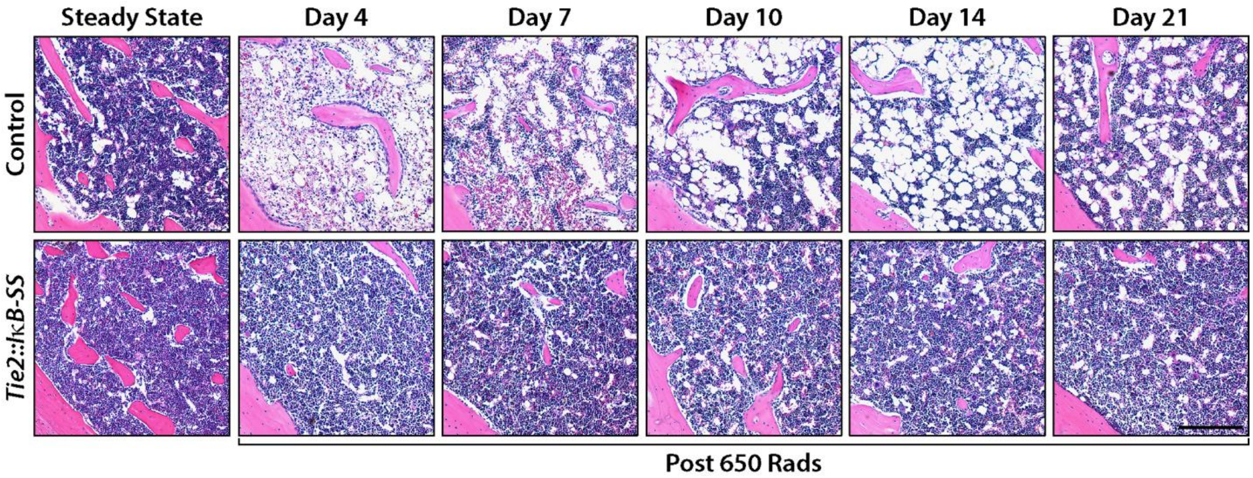

Supplementary Figure 5. Endothelial NF-κB inhibition protects bone marrow following irradiation. Time-course of representative hematoxylin and eosin (H&E) stained sections of the bone marrow following a myelosuppressive dose of total body irradiation (650 Rads). Scale bar = 500 μm.

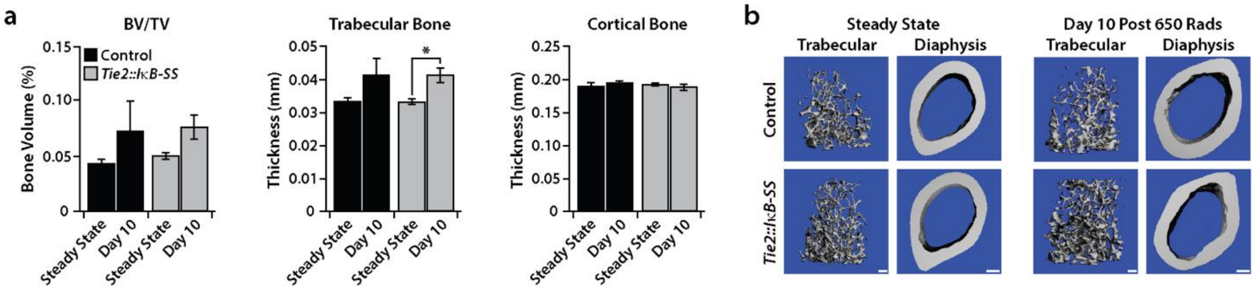

Supplementary Figure 6. ***Tie2::IκB-SS*** **mice display no overt changes in bone morphology at homeostasis or during regeneration.** Adult *Tie2::IκB-SS* and littermate control mice (12-16 weeks) were subjected to 650 Rads and assessed for femur morphology ten days post-injury. **a,** Micro-computed tomography (μCT) quantification of trabecular and cortical morphology (BV/TV = bone volume/total volume) (n = 4, steady state control; n = 4, day 10 control; n = 5, steady state *Tie2::IκB-SS*; n = 6, day 10 *Tie2::IκB-SS*). Error bars represent mean ± SEM. *P<0.05, **P<0.01, ***P<0.001. Pairwise comparisons were performed using Student's t-test; biological replicates. **b,** Representative three-dimensional reconstructions of femoral μCT scans are shown. Scale bars = 200 μm**.**

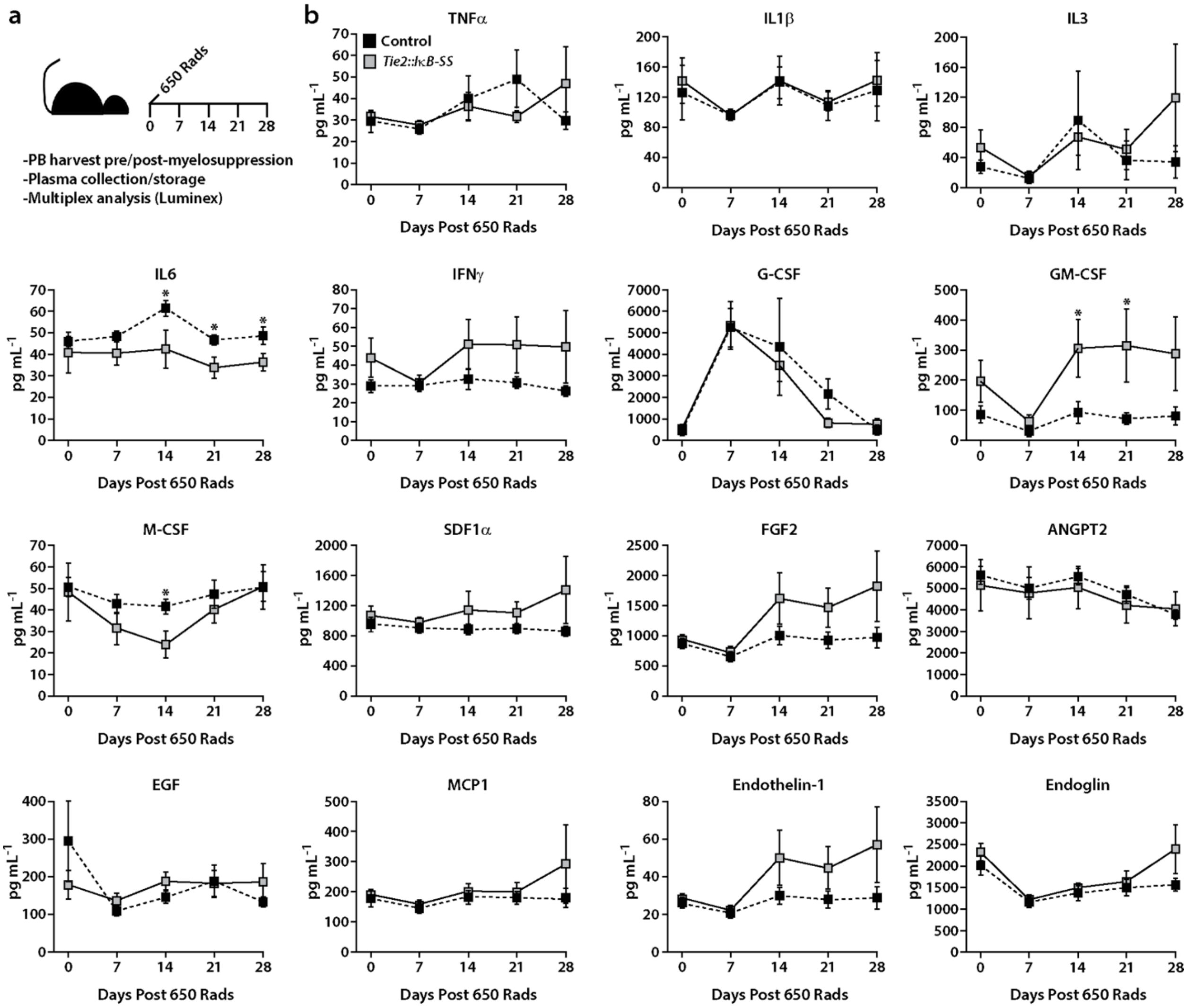

Supplementary Figure 7. ***Tie2::IκB-SS*** **mice display an increase in peripheral levels of GM-CSF during hematopoietic recovery.** **a,** Schematic of peripheral blood cytokine time-course following myelosuppressive irradiation. Peripheral blood plasma was isolated from control and *Tie2::IκB-SS* mice at steady state (Day 0) and following a sublethal dose of radiation (650 Rads) and **(b)** concentrations were assessed at 7 day intervals. Error bars represent mean ± SEM. *P<0.05, **P<0.01, ***P<0.001. Pairwise comparisons were performed using Student's t-test; biological replicates.

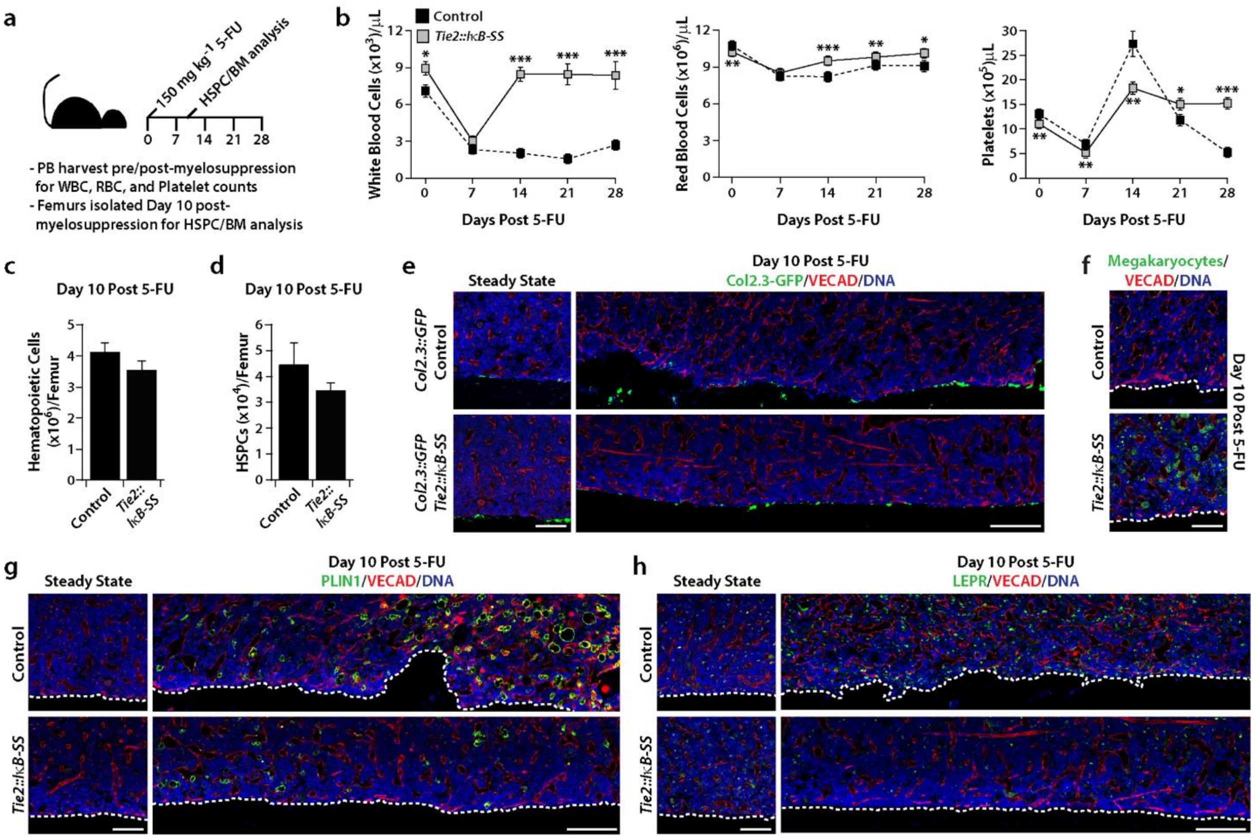

Supplementary Figure 8. Endothelial-specific inhibition of NF-κB signaling enhances hematopoietic recovery following chemotherapeutic injury. Adult *Tie2::IκB-SS* and littermate control mice (12-16 weeks) were subjected to a single dose of 150 mg/kg 5-fluorouracil (5-FU) and assessed for hematopoietic recovery. **a,** Schematic of 5-FU administration and peripheral blood (PB)/bone marrow (BM) analysis. **b,** Time-course of hematopoietic recovery in peripheral blood (n = 22, control; 18, *Tie2::IκB-SS*). **c-d,** Quantification of total hematopoietic cells **(c)** and phenotypic hematopoietic stem and progenitor cells **(d)** (HSPCs; cKIT^{+}Lineage^{-}SCA1^{+}) (n = 10, control; 8, *Tie2::IκB-SS*). **e**, Representative images of femurs from *Col2.3::GFP; Tie2::IκB-SS* and *Col2.3::GFP* control mice at steady state and following myelosuppression. Intravitally labeled vasculature (VECAD; red), *Col1a1* expressing osteoblasts (green), and nuclear staining (DAPI; blue) is noted. Scale bar = 100 μm and 200 μm, respectively. **f,** Representative images of intravitally labeled vasculature (VECAD; red), megakaryocytes (green; citrulline) and nuclear staining (DAPI; blue). Dashed line demarcates bone. Scale bar = 100 μm. **g-h,** Representative images of femurs stained with antibodies raised against PLIN1 (**g;** green) or LEPR (**h;** green), intravitally labeled vasculature (VECAD; red), and nuclear staining (DAPI; blue). Dashed line demarcates bone. Scale bar = 100 μm and 200 μm, respectively. Error bars represent mean ± SEM. *P<0.05, **P<0.01, ***P<0.001. Pairwise comparisons were performed using Student’s t-test; biological replicates.

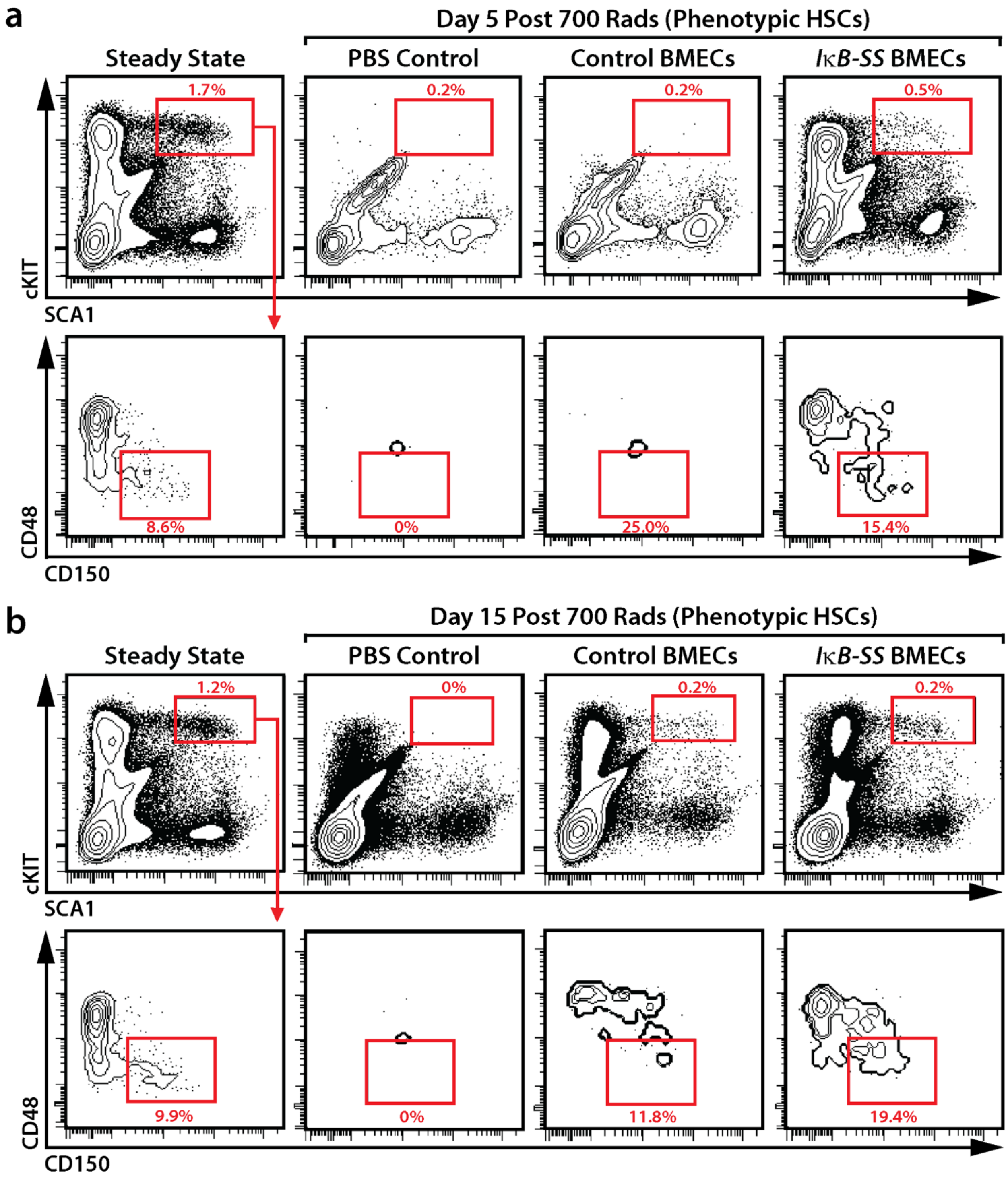

Supplementary Figure 9. ***Tie2::IκB-SS*** **BMEC mitigates HSC loss following radiation-induced myeloablation. a-b,** Representative contour flow plots of phenotypic hematopoietic stem cells (HSCs; $cKIT^{+}Lineage^{-}SCA1^{+}CD150^{+}CD48^{-}$) from whole bone marrow at day 5 **(a)** and day 15 **(b)** post-irradiation.

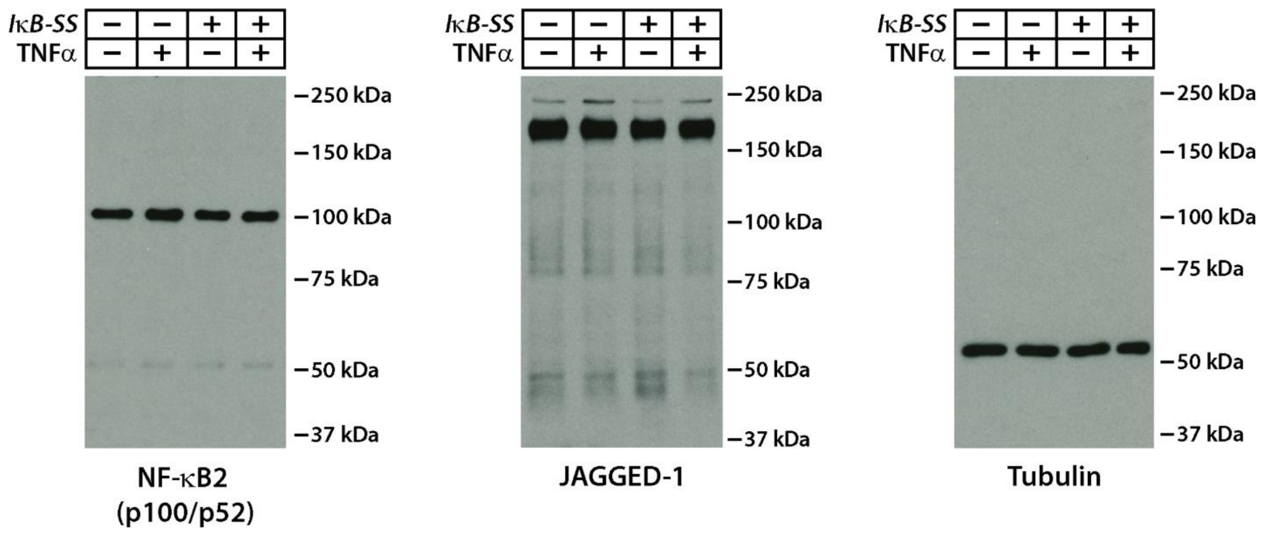

Supplementary Figure 10. Uncropped NF-κB2 and JAGGED-1 Western blots. Representative uncropped Western blots. Note: NF-κB2 (Supplementary Fig. 1i) and JAGGED-1 (Supplementary Fig. 2c) blots share a common Tubulin loading control.
